# Calibration-free physics-informed multi-task residual U-Net for simultaneous denoising and gas pressure retrieval from noisy voigt spectra

**DOI:** 10.1038/s41598-026-57932-1

**Published:** 2026-06-13

**Authors:** Alireza Raheemi Bahambari, Alireza Khorsandi

**Affiliations:** Department of Physics, University of Isfahan, Hezar Jarib Street, Isfahan, 81746- 73441 Iran

**Keywords:** Engineering, Mathematics and computing, Physics

## Abstract

We present a machine learning framework based on a one-dimensional U-Net (1D U-Net) that simultaneously performs spectral denoising and pressure estimation within a unified architecture. The model is trained on simulated Voigt profiles of the P(21) CO absorption line over pressures ranging from of 1 mbar to 2 bar. To ensure realistic conditions, simulated spectra are superimposed with experimentally captured noise, making them nearly indistinguishable from real experimental scans and forcing the model to recover clean spectra from noisy inputs. Quantitative assessments indicate excellent reconstruction performance, with a Pearson correlation coefficient (PCC) approaching unity, a signal-to-noise ratio (SNR) exceeding 35 dB, and both mean absolute error (MAE) and mean squared error (MSE) remaining close to zero. The model accurately predicts pressures for 200 unseen spectra using only spectral features, bypassing traditional linewidth analysis. At 1.25 bar, the U-Net yields virtually zero error (AE ≈ 0, SE ≈ 0), demonstrating sub-percent deviation and excellent consistency with the simulated reference. Experimental validation on a difference-frequency generation (DFG) spectrometer confirms robust performance, with ≈ 70% of traces reaching a peak SNR (PSNR) above 34 dB. Pressure estimation from ramp-based scans further demonstrates high accuracy, achieving minimal errors at 539.1 mbar (AEP = 0.002, SEP = 4.0 × 10⁻⁶). These findings establish the 1D U-Net as an efficient and reliable alternative to conventional noise-reduction and pressure-estimation techniques, simplifying mid-infrared spectroscopy workflows while ensuring high fidelity and stability.

## Introduction

 Laser absorption spectroscopy (LAS) is a widely used technique for trace gas detection due to its high selectivity and sensitivity, with detection limits ranging from ppm to ppb^1^. Advances in mid-infrared (MIR) laser sources, particularly quantum cascade (QC) lasers, have significantly expanded the applicability of LAS in environmental monitoring, medical diagnostics, and chemical analysis^2–4^. For instance, a QC-laser-based absorption sensor enabled simultaneous detection of SO₂ and SO₃ at elevated temperatures, achieving minimum detection limits of 8 ppb for SO₂ and 3 ppb for SO₃ with a 100 s averaging time, while enabling real-time monitoring of SO₂ oxidation by O₃ at 460 K^5^. In addition, distributed feedback (DFB) MIR lasers, characterized by linewidths below 2 MHz and milliwatt-level output power, provide stable spectroscopic measurements for sensitive gas detection. Their performance has been demonstrated for CO detection near 4.58 μm, where the use of a hollow waveguide with an optical loss of 0.73 dB further improved detection sensitivity^6^.

Recent developments in difference-frequency generation (DFG) via random quasi-phase-matching (RQPM) in polycrystalline Cr: ZnSe enabled the production of ultrabroad nanosecond MIR pulses spanning 7.6–16 μm for sensitive N₂O detection^7^. The intensity noise of DFG sources exhibits pulse-to-pulse stability of 0.6–1.9% RMS across the 3.5–8 μm spectral range, primarily determined by pump gain and signal noise interactions^8^. Although this noise level is slightly higher than that of self-phase-locked optical parametric oscillators (OPOs)^9^, DFG systems offer broader spectral coverage and a simpler optical architecture, making them attractive for broadband MIR spectroscopy.

Phase-sensitive detection (PSD) combined with MIR-DFG spectroscopy significantly improves the signal-to-noise ratio (SNR) and sensitivity, providing stability against environmental fluctuations essential for high-precision measurements^10^. Similarly, wavelength modulation spectroscopy (WMS) has been widely applied in MIR systems; for example, DFG-based WMS at 3.87 μm achieved an N₂O detection limit of 150 ppbv in a 29.9 m Herriott cell at 12 kPa^11^. Despite its effectiveness, WMS presents several limitations, including susceptibility to optical interference fringes, signal distortion under noisy conditions, and residual amplitude modulation (RAM) noise, which collectively degrade detection accuracy and sensitivity^12–14^. Moreover, modulation and demodulation processes may suppress fine spectral features, potentially discarding useful absorption information. These limitations, together with the complexity of modulation-based signal processing, motivate the use of simpler direct-absorption approaches^15^. In practical spectroscopic sensing systems, measurement data are often affected by noise, environmental fluctuations, and instrumental instability, which makes effective denoising and feature extraction essential for achieving accurate spectral interpretation and reliable pressure estimation^2^.

Machine learning (ML) has recently emerged as an effective tool for improving spectral analysis under noisy conditions. Techniques such as principal component analysis (PCA) and regression models including extreme learning machines (ELM) have been used to enhance signal recovery and noise suppression. For example, a semi-supervised PCA-based filter improved the signal-to-noise ratio of ro-vibrational spectra measured by QCL-based MIR cavity ring-down spectroscopy (CRDS)^16^. Another study combined ELM with an ELM autoencoder (ELM-AE) to perform simultaneous feature extraction and regression for quantitative analysis of gas mixtures, outperforming PCA-based and partial least squares methods on simulated and experimental infrared spectra of N₂O, NO₂, and NO^17^.

Despite these advances, most ML approaches treat spectral denoising and regression as separate tasks. Recent ML approaches effectively address spectral peak overlaps and improve quantitative analysis, enabling real-time, high-precision monitoring. Extracting physical parameters (pressure, gas concentration) from ML-processed signals requires careful calibration, but this calibration is performed only once against a reference system. Once calibrated, the ML model operates reliably without further adjustments, making it practical for field-deployed spectroscopic systems where continuous recalibration is infeasible. Traditional analysis methods rely on iterative Voigt profile fitting, which can provide accurate results but becomes computationally expensive and unstable for noisy spectra. In practice, peak-analysis software such as PeakFit^18^ is often used to automate nonlinear fitting, although the process remains time-consuming and operator dependent despite the availability of alternative packages^19,20^.

Recent studies suggest that ML models can recover absorption features from noisy spectra and enable reliable extraction of parameters such as pressure or gas concentration. These approaches have also been reported to outperform conventional denoising techniques, including discrete wavelet transform (DWT) and adaptive neuro-fuzzy inference systems (ANFIS)^21^. More recently, a ResNet-based model was proposed to directly estimate gas pressure from spectral line images, bypassing traditional peak fitting. In simulations involving 200 samples, the model achieved MAE = 0.095 and MSE = 0.009. Experimental validation using 13 samples below 94 mbar reported a maximum MAE of 1.2 × 10⁻² and MSE of 1.46 × 10^-4^^22^, although the limited experimental dataset constrains evaluation of real-world performance.

In this work, we propose a multi-task deep learning framework that integrates spectral denoising and gas-pressure estimation within a single architecture. The proposed approach employs a unified one-dimensional U-Net (1D U-Net) model that performs both tasks simultaneously in a single forward pass. The network is trained on simulated spectra of the P(21) CO absorption line over a range of pressures and later fine-tuned using experimental data while maintaining the same architecture.

Synthetic spectra are combined with experimentally measured noise to generate realistic signals in which the absorption features are partially obscured. The trained model reconstructs the underlying absorption profiles and directly estimates gas pressure from the spectral features. The framework is evaluated using both simulated datasets and experimental pressure-dependent spectra measured with a DFG-based spectrometer, where the predicted pressures show strong agreement with the reference values.

The key contribution of this study is the direct estimation of gas pressure from denoised spectral signals without requiring background correction or ramp-voltage-to-wavenumber calibration. Unlike the image-based approach reported in^22^, the proposed method operates directly on spectral data, enabling robust denoising and pressure estimation while simplifying the overall spectroscopic workflow.

## Methods

### 1D U-Net model capability in denoising and pressure estimation: simulation

The objective of this study is twofold: (i) to recover the P(21) CO absorption line from a noisy spectrum in which the feature is deeply buried, and; (ii) to estimate gas pressure directly from the recovered spectral profile without relying on conventional peak-fitting methods such as PeakFit. To accomplish these tasks, a multi-task learning framework was developed that jointly performs spectral denoising and pressure estimation within a single neural architecture.

**Fig. 1:**
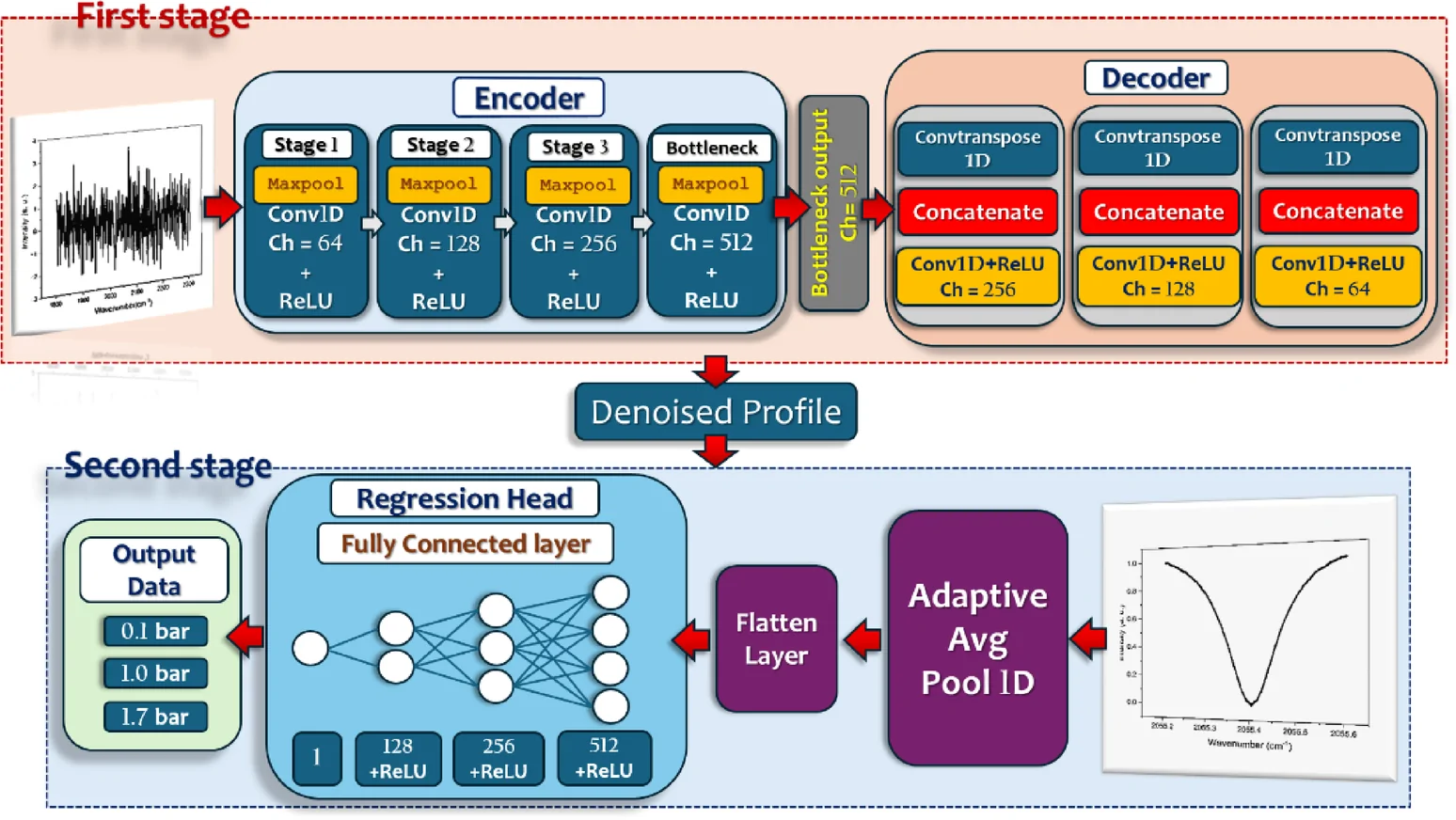
Two-stage deep learning pipeline for Voigt profile analysis and gas pressure prediction. First stage: A 1D U-Net model denoises the input spectrum noise using Conv1D-ReLU-MaxPool encoder blocks and ConvTranspose1D decoder layers with skip connections. Second stage: A lightweight regression network applies adaptive average pooling and a fully connected layer to predict normalized pressure.

The proposed model is based on a 1D U-Net architecture augmented with residual blocks at each encoder and decoder stage. This design enables simultaneous signal recovery and pressure estimation by leveraging deep feature extraction and multi-scale processing. A schematic overview of the model architecture is shown in Fig. 1.

As illustrated in the schematic shown in Fig. 1, the proposed model is a multi-task architecture built upon a 1D U-Net backbone. The network receives a two-channel input composed of the noisy spectrum and its corresponding wavenumber array, which is processed through a series of convolutional encoder-decoder blocks with ReLU activations. The denoising branch outputs a reconstructed spectrum that preserves the underlying Voigt lineshape.

In addition to signal reconstruction, the network includes a pressure estimation branch implemented as a lightweight regression head. Both tasks share the same encoder-decoder backbone, enabling joint feature learning within a unified architecture. The pressure estimation module operates on the reconstructed spectrum generated by the denoising branch.

Specifically, the reconstructed spectrum is processed by a compact regression module composed of adaptive average pooling, a flattening layer, and fully connected layers that produce a scalar pressure prediction. During inference, the model first reconstructs the denoised spectral profile from the noisy input and subsequently estimates the corresponding gas pressure within the range of 0.01–2.2 bar.

Model training was conducted using a 5-fold cross-validation strategy. The dataset was divided into five folds, where in each iteration four folds were used for training and the remaining fold was used for validation. To prevent information leakage between samples generated under identical physical conditions, a group-based splitting strategy was employed, ensuring that spectra corresponding to the same pressure value were assigned to the same learning rate of 3 × 10⁻⁴ and weight decay of 1 × 10⁻⁴. Early stopping with a patience of seven epochs was applied within each cross-validation fold. In addition, a ReduceLROnPlateau scheduler was used to decrease the learning rate when the validation loss stopped improving.

Each spectrum was normalized using per-sample mean-variance normalization. Data augmentation was applied to the noisy spectra and included additive Gaussian noise, uniform noise, and low-amplitude sinusoidal perturbations to improve model robustness. The multi-task loss function combined the denoising mean squared error (MSE) and the pressure regression MSE, with the regression loss weighted by a factor of 0.2. Model performance was monitored using the validation loss and the peak signal-to-noise ratio (PSNR) computed from the reconstruction error.

All computations were performed using a PyTorch implementation on a workstation equipped with an AMD Radeon GPU (2 GB VRAM) and a 7th-generation 5-core CPU. Our training dataset consisted of 2000 synthetic Voigt absorption lineshapes simulated for the CO P(21) transition at 2055.400 cm⁻¹, covering pressures uniformly distributed in the range 0.01–2.2 bar. To improve model generalization, each clean spectrum was used to generate four noisy realizations, resulting in 10,000 total samples.

The Voigt profile, which models spectral lines with both Doppler and pressure broadening, is mathematically expressed as:

1 $$\varphi _{V} \left( {\bar{\nu } - \bar{\nu }_{0} } \right) = \varphi _{D} \left( {\bar{\nu } - \bar{\nu }_{0} } \right)_{{\bar{\nu } = \bar{\nu }_{0} }} V\left( {a,w} \right),$$

where $\phi_{D} (\bar{\nu} - \bar{\nu}_{0} )$is the Doppler lineshape given by:

2 $$\varphi _{D} \left( {\bar{\nu } - \bar{\nu }_{0} } \right) = \frac{1}{{{{\Delta }}\bar{\nu }_{D} }}\left[ {\frac{{ln2}}{\pi }} \right]^{{1/2}} exp\left[ { - ln2\left( {\frac{{\bar{\nu } - \bar{\nu }_{0} }}{{{{\Delta }}\bar{\nu }_{D} }}} \right)^{2} } \right].$$

In Eq. (1), the Voigt function *V(a,w)*describes the spectral line shape. The parameter *a* represents the Lorentzian broadening component, which varies with gas pressure, while *w*denotes the normalized frequency displacement relative to the line center ${\nu }_{0}$.

The Doppler width ${\Delta }{\nu }_{D}$, determined by the ambient temperature *T* and the CO molecular mass *M*_CO_, was held constant at 0.00480 cm⁻¹ (FWHM) under standard conditions. In contrast, the Lorentzian width varies linearly with pressure according to parameters obtained from the HITRAN (High-Resolution Transmission Molecular Absorption) database.

For the P(21) transition, the self-broadening coefficient was taken as *γ*_self_ = 0.053 cm^− 1^ atm^− 1^. Accordingly, the Lorentzian half-width was calculated as ${\gamma }_{L}={\gamma }_{\mathrm{self}}{P}_{\mathrm{C}\mathrm{O}}$ where ${P}_{\mathrm{C}\mathrm{O}}$ denotes the absolute pressure of CO gas. The resultant Voigt profile emerges from the convolution of these Doppler and Lorentzian contributions, defining the composite linewidth as described by^23^:

3 $${{\Delta }}\bar{\nu }_{V} = 0.5346{{\Delta }}\bar{\nu }_{L} + \sqrt {0.2166{{\Delta }}\bar{\nu }_{L} ^{2} + ~{{\Delta }}\bar{\nu }_{D} ^{2} } .$$

Using the mathematical formulations and data obtained from the HITRAN database, the absorption line corresponding to the CO P(21) transition was simulated. The Voigt spectra were generated by combining the Gaussian and Lorentzian components using the complex error function (wofz) implemented in the SciPy library. The wavenumber axis spans ± 0.2 cm^− 1^ around a center randomly shifted by ± 0.005 cm^−1^ to mimic small frequency drifts. Spectral profiles were normalized to unity peak intensity. Additional intensity and wavenumber augmentations were applied to simulate realistic variations in frequency and amplitude.

To ensure robustness across both theoretical simulations and practical applications, the proposed framework adopts a two-stage training strategy. In the first stage, the model is pretrained on synthetic Voigt spectra spanning pressures from 0.01 to 2.2 bar, allowing it to learn the general relationship between spectral features and gas pressure. This pretraining stage is not intended to re-estimate known spectroscopic parameters; rather, it enables supervised learning of the inverse mapping from spectral features to physical quantities, thereby providing a physics-informed initialization for the subsequent training stage. In the second stage, domain-adaptation fine-tuning is performed using pseudo-experimental data that reproduce the characteristic DFG sawtooth ramp slope and noise patterns observed in the measurement system. Importantly, this process is conducted without exposure to the test dataset.

The fine-tuning stage aligns the general, physics-informed representation learned during pretraining with the characteristics of the specific experimental setup, enabling improved practical performance without retraining the model from scratch or sacrificing generalization capability.

While the base model was trained on idealized Voigt profiles, experimental traces typically contain system-specific artifacts, most notably baseline drift induced by the DFG sawtooth ramp. To mitigate this domain shift, the model was fine-tuned using a separate pseudo-experimental dataset comprising approximately 175 synthetically generated samples designed to reproduce the ramp slope, baseline distortion, and noise characteristics of the DFG system. Importantly, the 16 experimental traces reported in the Results section were not used during either the pretraining or fine-tuning stages, and were reserved exclusively for model evaluation.

During the adaptation stage, only the pressure regression head was updated, while the denoising module and shared feature-extraction layers were kept fixed. This selective fine-tuning reduces the risk of overfitting to setup-specific artifacts and preserves the physics-informed representations learned during large-scale simulation pretraining.

The model was subsequently evaluated exclusively on the 16 unseen experimental traces, ensuring that the reported performance reflects true generalization rather than memorization of baseline drift or noise patterns specific to the experimental setup. During the inference phase, the system operates according to the type of input data specified by the user. When the input is identified as experimental, the model automatically loads the fine-tuned weights. In this configuration, the network processes raw, unprocessed signals acquired from the experimental setup, where the signal domain is defined by the DFG sawtooth ramp voltage. This enables the model to compensate for the associated baseline slope and hardware-specific fluctuations.

Conversely, when the input is specified as simulated, the base model weights are used. In this mode, the spectra are defined in the standard wavenumber domain with a flat baseline, corresponding to ideal Voigt profiles generated without ramp-induced artifacts.

## Results

### Denoising procedure

Four distinct noise signatures extracted from experimental spectroscopic traces were RMS-normalized and resampled to a uniform length of 1000 points. The ramp-generated noise spectrum was acquired experimentally as datasets while the phase-sensitive detection system comprising a lock-in amplifier and mechanical chopper along with the CO-filled absorption cell, were excluded from the DFG setup described in the following section.

The extracted noise profiles were added to the simulated spectra using randomly assigned signal-to-noise ratios (SNR) ranging from 0.02 to 0.2, together with additional Gaussian noise (σ = 0.01). All augmentation parameters were documented to ensure reproducibility. By overlaying these experimentally derived noise signatures onto the simulated P(21) CO spectra, the resulting signals became visually indistinguishable from noise, closely replicating realistic measurement conditions in the absence of noise-suppression techniques such as phase-sensitive detection.

**Fig. 2:**
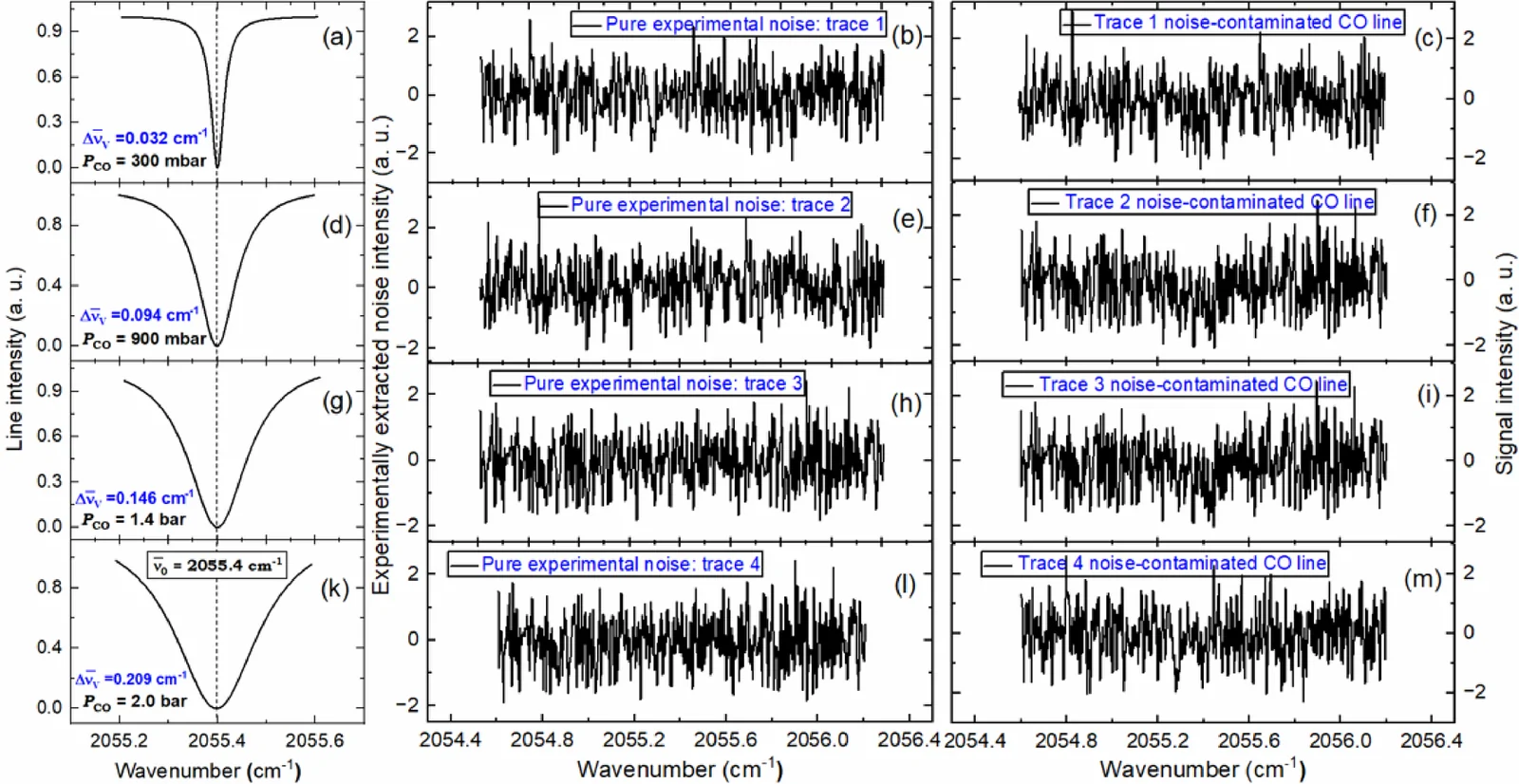
Simulated P(21) CO Voigt spectra at four gas pressures (left), experimentally measured pure noise spectra recorded after removal of the CO absorption cell and phase-sensitive detection components (middle), and the resulting noise-contaminated CO signals obtained by combining the two (right). The noise amplitude was high enough to fully obscure the simulated absorption lines. The inset in the left panels shows the Voigt linewidths from Eq. (3), where the Doppler width was kept constant and the Lorentzian width varied with gas pressure.

The dataset was organized into paired clean and noisy CSV files, with metadata linking each noisy spectrum to its corresponding clean reference, enabling the supervised two-stage training pipeline. After training the 1D U-Net, an additional 200 independent simulated samples were generated to evaluate model robustness. These samples were not used during training. Specifically, 200 Voigt profiles spanning pressures from 0.01 bar to 2 bar were synthesized and processed by the denoising network. Figure 2 illustrates four simulated CO absorption spectra at different pressures, each corrupted by distinct experimentally extracted noise sequences. The figure also includes an example in which the CO absorption signal is completely submerged in noise, making it indistinguishable prior to denoising.

**Fig. 3:**
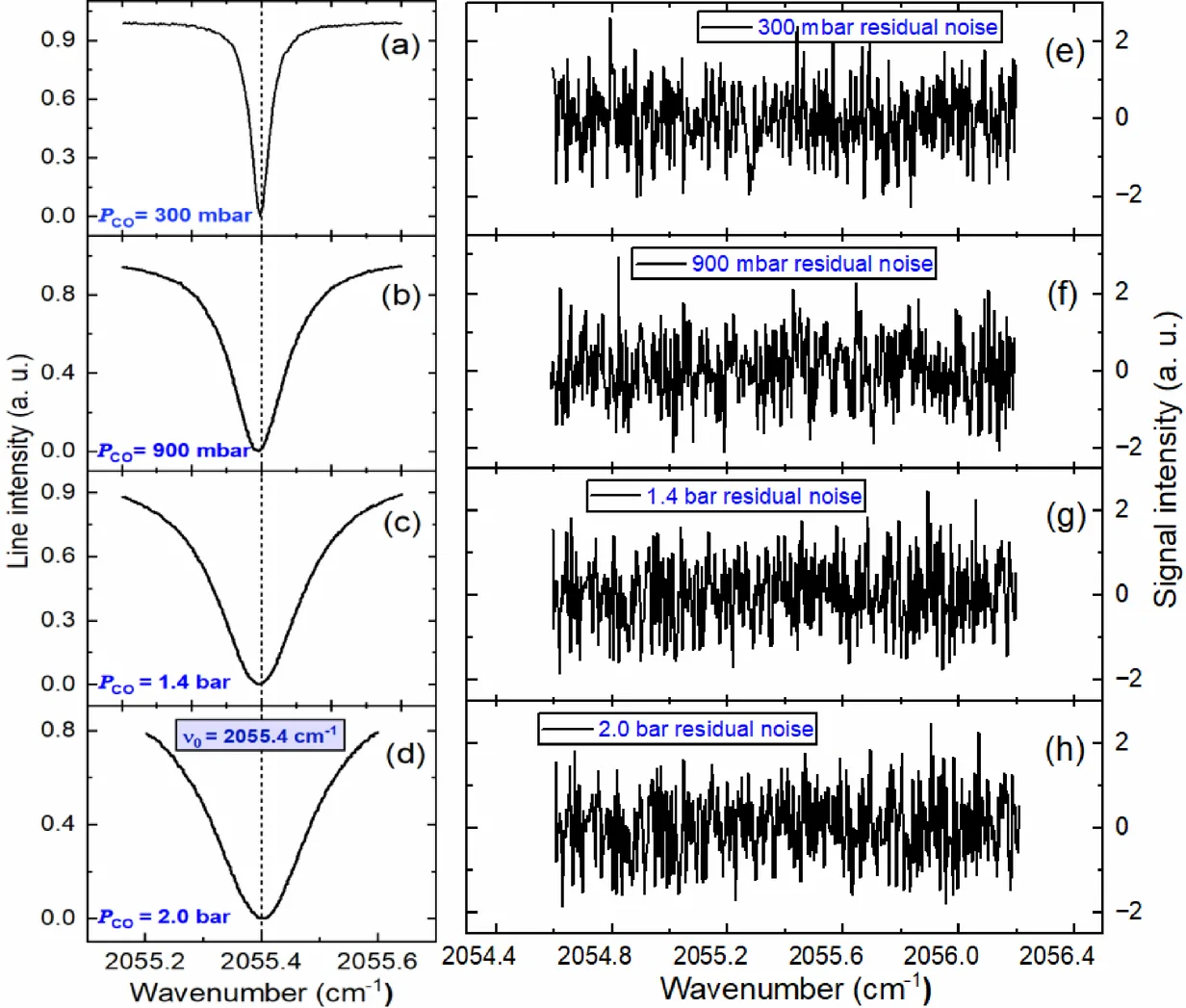
Qualitative denoising performance of the 1D U-Net model. Plots (**a**) to (**d**) show the ML-extracted spectra of the P(21) CO line after being extracted from spectra contaminated with four traces of experimental noise. Plots (**e**) to (**h**) display the corresponding residual noise following 1D U-Net-based denoising.

As shown in Fig. 2, the experimentally extracted noise spectra labeled (b), (e), (h), and (l) Exhibit fluctuations that are on average about 3.6 times greater than the absorption depths of the corresponding simulated CO lines presented in panels (a), (d), (g), and (k). To rigorously assess the denoising model introduced in Fig. 1, the simulated clean Voigt profiles were embedded entirely within an experimental noise floor of sufficient magnitude, make the features fully indistinguishable. Representative samples displayed in plots (c), (f), (i), and (m), selected from the 200 test profiles independent of the training dataset, clearly demonstrate that the CO lines are completely hidden within the noise. This stringent evaluation setup provides a robust test of the 1D U-Net’s ability to reconstruct weak CO absorption features from severely degraded signals. The model’s denoising performance is further demonstrated in Fig. 3.

As can be seen in Fig. 3, the model effectively recovers the P(21) CO spectrum from the severely noise-contaminated spectra shown in Fig. 2c, f, i, m. This qualitative agreement with the simulated spectra supports the effectiveness of the signal-processing pipeline illustrated in Fig. 1, indicating that the ML-based approach remains reliable even under strong noise conditions.

**Fig. 4:**
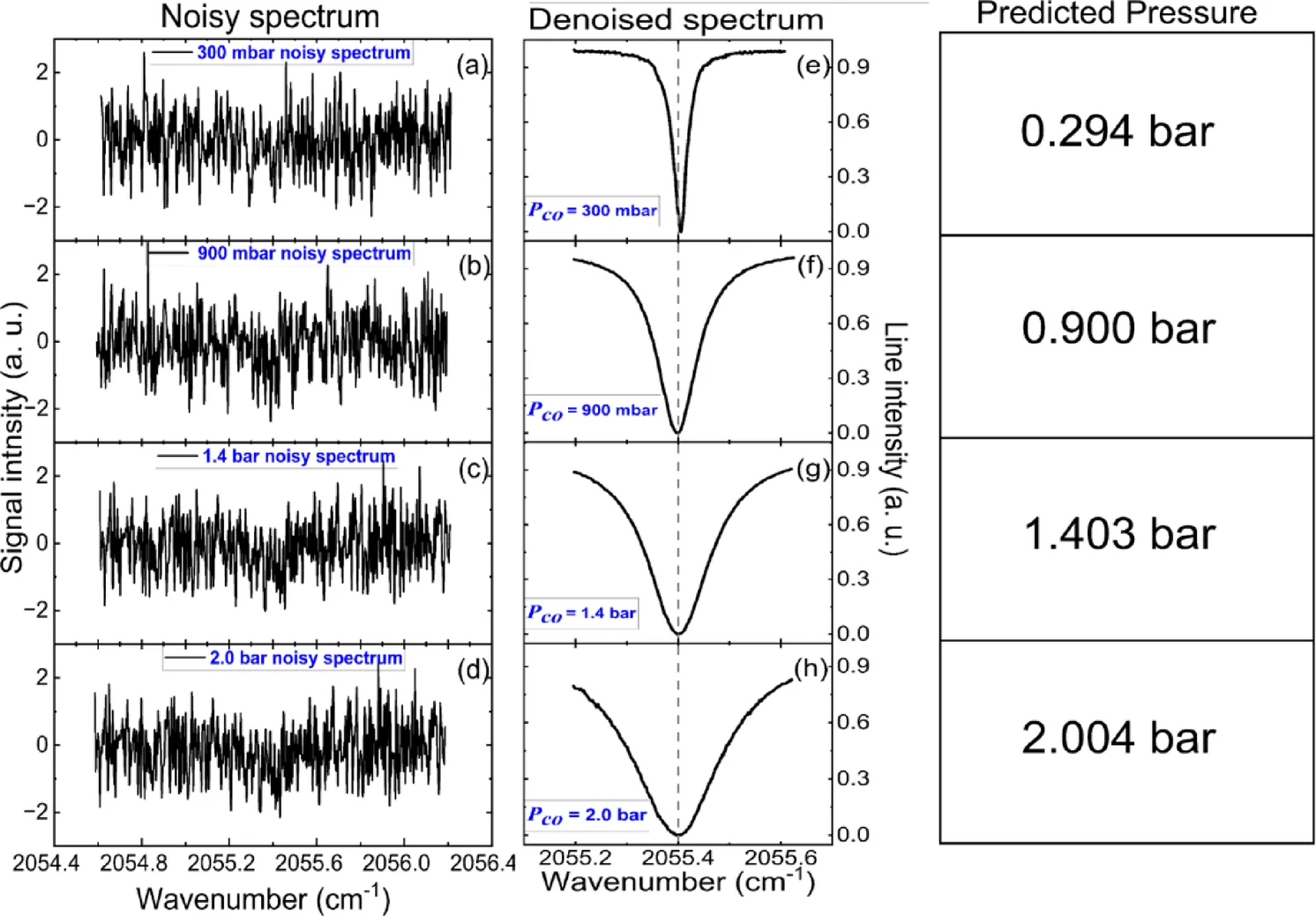
Noisy input spectra for (**a**) 0.3 bar, (**b**) 0.9 bar, (**c**) 1.4 bar, and (**d**) 2.0 bar, reconstructed denoised spectra (**e**) to (**h**), and predicted pressure values (right panel) obtained from the proposed model.

A small shift of approximately ±0.02 cm⁻¹ from the line center is observed when comparing the reconstructed spectra with the corresponding simulated profiles in Fig. 2a, d, g, k. Nevertheless, their line shapes and widths remain remarkably consistent. Figure 4 presents the model pipeline. A noisy spectrum is given as input, the network reconstructs a denoised spectrum, and the corresponding pressure is predicted directly from the same input.

Even under strong noise, the model recovers smooth line shapes and provides accurate pressure estimates. The results demonstrate stable joint denoising and parameter prediction within a single framework. To quantitatively evaluate the effectiveness of the denoising model, we employed five complementary metrics as follows:

- Signal-to-Noise Ratio (SNR), defines as^24^.

4 $$SNR \left(dB\right)=10lo{g}_{10}\left(\frac{{\sum }_{i=1}^{N}{x}_{i}^{2}}{{\sum }_{i=1}^{N}{\left({x}_{i}-{\widehat{x}}_{i}\right)}^{2}}\right),$$

where ${x}_{i}$ and ${\widehat{x}}_{i}$ denote the clean reference and denoised signals, respectively, and *N*is the number of samples. SNR measures the ratio of the clean signal power to the residual noise in the reconstructed output. The clean reference is the noise-free simulated CO spectrum (Fig. 2), and the denoised signal is the model output (Fig. 3). Signal and noise powers are computed as the sums of squared amplitudes of the clean signal and the clean–denoised difference, respectively.

- Mean Squared Error (MSE), which is one of the most fundamental metrics for quantifying the magnitude of the error between two signals, is given by^25^.

5 $$MSE=\frac{1}{N}\sum\limits_{i=1}^{N}{\left({x}_{i}-\widehat{{x}_{i}}\right)}^{2}.$$

The MSE quantifies the average squared difference between the clean reference signal and the denoised output. By squaring the errors, MSE emphasizes larger deviations, effectively penalizing outliers. A lower MSE indicates higher denoising accuracy and better signal reconstruction fidelity.

- Mean Absolute Error (MAE), that is^25^.

6 $$\mathrm{M}\mathrm{AE}=\frac{1}{N}\sum\limits_{i=1}^{N}\left|{x}_{i}-\widehat{{x}_{i}}\right|,$$

provides a more uniform treatment of all errors without over-penalizing large deviations. It reflects the average magnitude of distortion and is more robust to outliers compared to MSE.

- Peak Signal-to-Noise Ratio (PSNR), that quantifies the fidelity of reconstructed or denoised data relative to the clean reference, with specific emphasis on preserving peak intensities, is expressed as^26^.

7 $$PSNR \left(dB\right)=10lo{g}_{10}\left(\frac{MA{X}^{2}}{MSE}\right),$$

where *MAX* denotes the maximum possible value of the clean signal, as is clear, a lower MSE leads to higher PSNR, indicating the reconstructed data closely matches the clean reference.

- Pearson’s Correlation Coefficient (PCC), which is a fundamental statistical metric, quantifies the linear relationship and structural similarity between the clean reference and denoised datasets respectively presented in Fig. 2 (a), (d), (g), and (k) and Fig. 3 (a) to (d). In the context of signal processing and denoising, it evaluates the extent to which the shape and relative changes in the denoised signal, $\widehat{{x}_{i}}$, mirror those in the clean reference signal, ${x}_{i}$. The PCC coefficient is^27^.

8 $$PCC=\frac{{\sum }_{i=1}^{N}\left({x}_{i}-\stackrel{-}{x}\right)\cdot \left(\widehat{{x}_{i}}-\overline{\widehat{x}}\right)}{\sqrt{{\sum }_{i=1}^{N}{\left({x}_{i}-\stackrel{-}{x}\right)}^{2}}\cdot \sqrt{{\sum }_{i=1}^{N}{\left(\widehat{{x}_{i}}-\overline{\widehat{x}}\right)}^{2}}},$$

where ${x}_{i}$ and ${\widehat{x}}_{i}$ denote the clean reference and denoised signals, $\stackrel{-}{x}$ and $\overline{\widehat{x}}$ also denote the mean values of them, respectively. An optimal denoising model yields a PCC close to unity. In practice, PCC > 0.95 indicates excellent structural similarity, confirming that the denoised signal’s shape, peaks, and trends closely match the clean reference, whereas PCC < 0.9 suggests reduced structural similarity and noticeable distortion of the signal’s fundamental shape. As a normalized metric, PCC isolates shape error from amplitude scaling, making it a reliable indicator of whether the model preserves the physical information encoded in the signal structure.

MSE and MAE quantify reconstruction error magnitude, while PCC evaluates structural similarity, ensuring preservation of the original waveform shape and proportionality—since low numerical error alone does not guarantee correct waveform reconstruction. SNR and PSNR further characterize noise suppression and peak fidelity. Table 1 quantifies the denoising performance of the 1D U-Net model for the spectra shown in Fig. 3. The model successfully reconstructs the CO absorption profiles from strongly fluctuating noise, even when the original signals are barely distinguishable.

**Table 1:** Standard metrics were employed to quantitatively assess the performance of the 1D U-Net model in reconstructing the denoised P(21) CO spectra shown in Fig. 3(a) to (d), derived from the exemplary noise-contaminated signals depicted in Fig. 2(c), (f), (i), and (m) and compared with the clean reference spectra in Fig. 2(a), (d), 9 g), and (k).

CO gas pressure (bar)	SNR (dB)	PSNR (dB)	MSE (⋅10^− 5^)	MAE (⋅10^− 3^)	PCC
0.3	41.46	41.67	6.80	7.29	0.9994
0.9	43.13	43.71	4.25	5.87	0.9998
1.4	45.49	46.38	2.30	4.06	0.9999
2.0	43.78	45.02	3.14	4.56	0.9997
3.0	41.25	42.13	2.05	2.94	0.9922

**Fig. 5:**
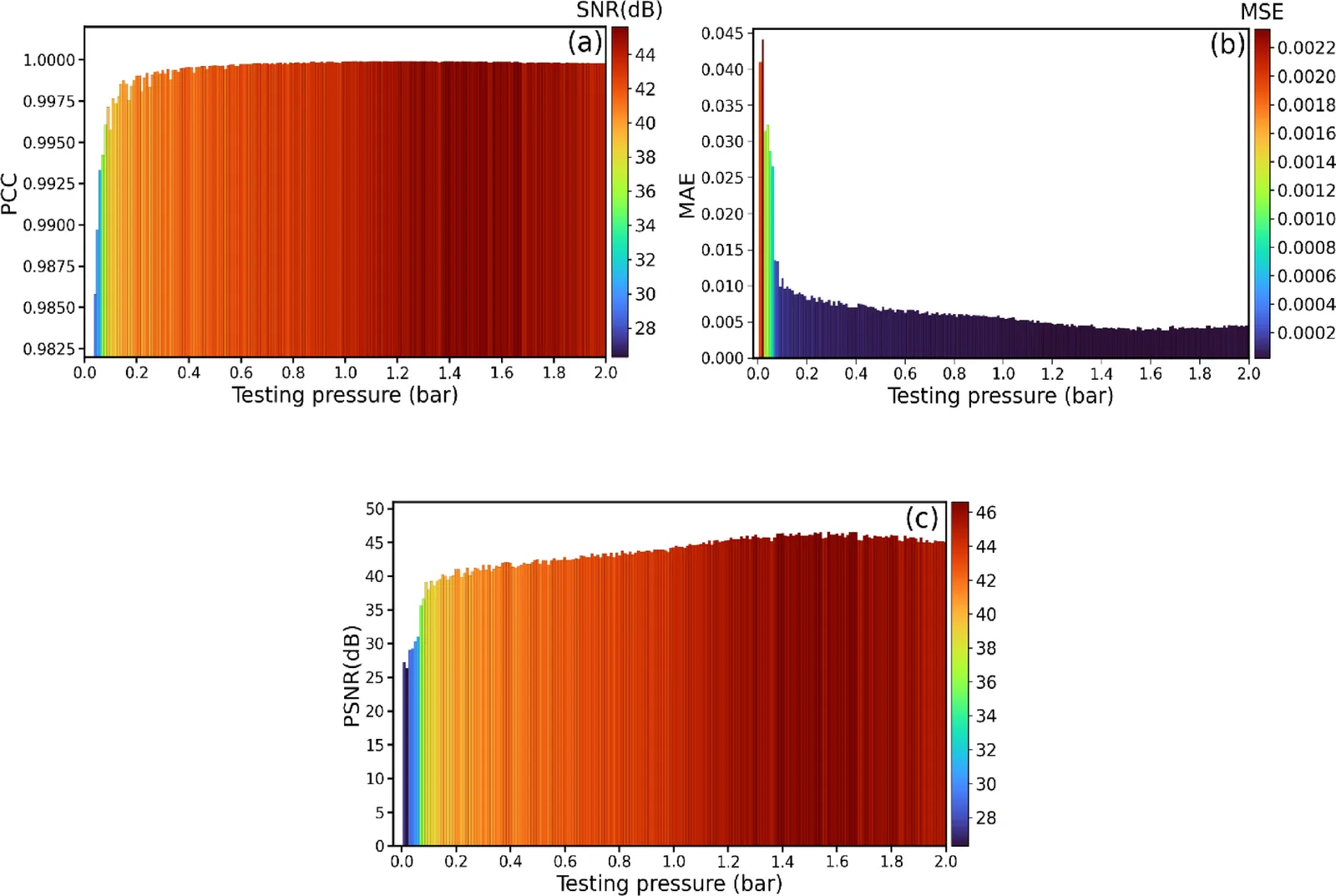
Scatter plots of the evaluated metrics across 200 simulated test pressures, demonstrating the denoising capability of the 1D U-Net model. (**a**) Displays the PCC with the SNR mapped to the color bar, (**b**) illustrates fluctuations in the MAE alongside the MSE, and (**c**) depicts variations in PSNR.

The results in Table 1 demonstrate that the 1D U-Net model effectively recovers CO spectra from highly fluctuating noise, where the original signals are nearly indistinguishable. As the CO pressure increases from 0.3 to 3.0 bar, both SNR and PSNR improve, reaching 43.78 dB and 45.02 dB at 1.4 bar, which corresponds to the best reconstruction performance. At the same time, MAE and MSE decrease, while PCC values remain close to unity, indicating strong agreement between the simulated and reconstructed spectra across all pressures. Compared with the previously used autoencoder^13^, the 1D U-Net achieves superior denoising and signal recovery under strong noise conditions. To further evaluate model performance, the distributions of the metrics for 200 simulated CO spectra not used for training are shown in the histogram in Fig. 5.

As shown in Fig. 5a, the SNR distribution is strongly skewed toward high values of (40–46 dB), indicating that the reconstructed signals exhibit a remarkably high SNR, with only a few instances below 35 dB. The SNR exhibits a monotonic increase from approximately 30 dB at low pressures to above 45 dB at around 1.4 bar, followed by a mild saturation. This trend implies that the model achieves stronger noise suppression when the physical signal becomes more pronounced at higher pressures. Similarly, the PCC remains consistently close to unity, exceeding 0.99 for almost all samples, indicating that the denoised outputs preserve the structural integrity and spatial correlation of the ground-truth signals. Specifically, PCC increases from approximately 0.998 at low pressure to nearly unity in the 1–2 bar range, confirming that the model maintains strong linear correspondence between reconstructed and reference signals across the entire pressure range. The PSNR variations in Fig. 5c follow a trend similar to the SNR behavior in Fig. 5a, although the performance degradation at low CO pressures is more pronounced over a wider range.

By contrast, as shown in Fig. 5b, the MSE and MAE distributions are tightly concentrated near zero, with most samples on the order of 10⁻³–10⁻⁴. This narrow, unimodal distribution reflects the numerical stability and convergence of the model across the test dataset. Both metrics decrease progressively with increasing pressure, dropping from about 2 × 10⁻⁴ at low pressures to an order of magnitude lower above 1 bar. The reduced denoising performance below 0.01 bar originates from the extremely narrow Voigt profiles that are easily obscured by background noise, making full line-shape recovery difficult. Despite these challenging low-pressure conditions, the denoised spectra remain highly correlated with the noise-free references, demonstrating that the model maintains both structural and spectral fidelity.

### Pressure estimation

**Fig. 6:**
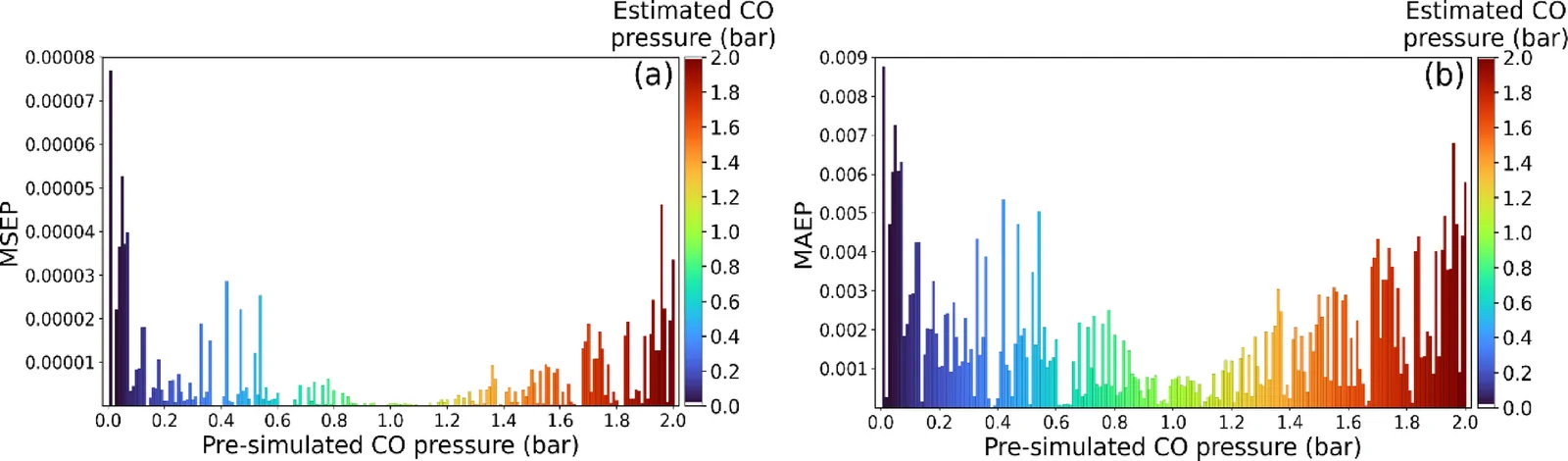
illustrates the MSEP and MAEP metrics in panels (a) and (b), respectively, as functions of simulated CO pressure, computed from Eqs. (9) and (10). These metrics assess how closely the pressures predicted by the second-stage 1D U-Net match the true simulated values across the 200 test samples. The color bar indicates the predicted pressures, with each vertical bar’s hue representing the agreement between simulated and estimated values. For instance, bars in the 1.8–2.0 bar range appear deep red, consistent with the corresponding color on the pressure scale.

The denoised P(21) CO spectra corresponding to 200 test pressure points were provided to the second stage of the architecture (Fig. 1) to evaluate gas pressure estimation accuracy, with particular emphasis on the reconstructed Voigt profiles. Using the same P(21) CO dataset (2000 simulated spectra), pressure was estimated from the denoised spectral profiles, and the predicted values closely agree with the ground-truth simulation pressures prior to noise addition (Fig. 6).

Unlike the previous study in Ref.^22^, where CO pressure was estimated from noise-free simulated Voigt spectral images, the present work estimates pressure directly from denoised CO spectra reconstructed from noisy signals by the first stage of the model (Fig. 1). The predicted pressures closely match the original simulation values prior to experimental noise addition. A more precise quantitative comparison is then performed using the mean absolute error (MAE) and mean squared error (MSE) metrics for pressure estimation, defined as:

9 $$MSEP=\frac{1}{N}\sum _{i=1}^{N}{\left({P}_{{CO}_{i}}^{Pre-simulated}-{P}_{{CO}_{i}}^{UNet1D-estimated}\right)}^{2},$$

and

10 $$MAEP=\frac{1}{N}\sum _{i=1}^{N}\left({P}_{{CO}_{i}}^{Pre-simulated}-{P}_{{CO}_{i}}^{UNet1D-estimated}\right),$$

Here, ${P}_{{CO}_{i}}^{Pre-simulated}$denotes the ground-truth clean CO pressure values (simulated prior to noise addition), while ${P}_{{CO}_{i}}^{UNet1D-estimated}$ refers to the pressure values inferred from the spectra denoised by the 1D U-Net model. *N* represents the total number of samples. A quantitative comparison between these references and estimated pressures is evaluated using MSEP and MAEP metrics, as shown in Fig. 6.

Figure 6 shows a pressure-dependent error trend: errors increase at very low pressures (≤ 0.05 bar), both MSEP and MAEP increase because weak optical absorption yields narrow Voigt profiles, making it difficult for the model to recover pressure-dependent features from noise. A secondary rise occurs near the upper limit (≈ 1.9-2.0 bar), mainly due to training constraints, where limited GPU precision and batch size slightly lower numerical resolution and reconstruction accuracy under sustained high-pressure conditions. outside these boundary regions, the model remains stable over 0–2 bar. The maximum errors of MAEP ≈ 7.26 × 10⁻³ and MSEP ≈ 7.70 × 10⁻⁵ at ≈ 0.05 bar, indicate that even under the weakest signal conditions, the network preserves strong quantitative accuracy.

It is important to note that the increased errors observed at very low pressures (< 0.05 bar) are primarily associated with physical limitations of the measurement rather than a failure of the learning model. In this regime, the pressure-broadening contribution remains extremely small compared with the Doppler component, resulting in a very narrow and weak absorption feature. Consequently, the pressure-dependent spectral variation becomes comparable to the experimental noise level.

Moreover, the intrinsic Voigt linewidth in this pressure range remains smaller than the laser linewidth, meaning that the spectrometer effectively observes a resolution-limited profile. As a result, part of the pressure information is lost through instrumental broadening. The combination of limited spectral resolution and low absorption contrast establishes a practical physical detection limit of the approach around ~ 0.05 bar. Below this threshold, pressure retrieval becomes predominantly constrained by instrumental resolution and noise floor rather than by the predictive capacity of the neural network.

To further illustrate the model’s performance, Table 2 reports the CO pressure estimates for the denoised spectra listed in Table 1, together with additional pressure values extracted from Fig. 5 for a more comprehensive evaluation.

**Table 2:** AEP and SEP values extracted from Fig. 5 for the example and additional CO pressures corresponding to Table 1.

Pre-simulated CO pressure (bar)	1D U-Net -estimated CO pressure (bar)	Error	
		AEP	SEP
0.01	0.0183	8.3 × 10^− 3^	6.8 × 10^− 5^
0.25	0.2496	4.0 × 10^− 4^	1.6 × 10^− 7^
0.30	0.3003	3.0 × 10^− 4^	9.0 × 10^− 8^
0.50	0.5003	3.0 × 10^− 4^	9.0 × 10^− 8^
0.75	0.7524	2.4 × 10^− 3^	5.7 × 10^− 6^
0.90	0.9053	5.3 × 10^− 3^	2.8 × 10^− 5^
1.00	1.0005	5.0 × 10^− 4^	2.5 × 10^− 7^
1.25	1.2500	0	0
1.40	1.4084	8.4 × 10^− 3^	7.0 × 10^− 5^
1.50	1.5091	9.1 × 10^− 3^	8.2 × 10^− 5^
1.75	1.7524	2.4 × 10^− 3^	5.7 × 10^− 6^
2.00	2.0131	1.3 × 10^− 2^	1.7 × 10^− 4^
2.50	2.5373	3.7 × 10^− 2^	1.3 × 10^− 3^
3.00	2.9459	5.4 × 10^− 2^	2.9 × 10^− 3^

The pre-simulated CO pressure represents the initial noisy input fed into the first stage of Fig. 1 for denoising, while the resulting denoised spectrum is subsequently processed in the second stage for pressure estimation. The gray-highlighted rows correspond to the examples listed in Table 1.

Table 2 reports the SEP and AEP values for pressure estimation. These metrics correspond to the squared error (SE) and absolute error (AE) computed for each individual pressure point, defined as:

11 $${\mathrm{S}\mathrm{E}\mathrm{P}}_{i}={\left({P}_{\mathrm{C}\mathrm{O},i}^{\mathrm{P}\mathrm{r}\mathrm{e}\mathrm{-}\mathrm{s}\mathrm{i}\mathrm{m}\mathrm{u}\mathrm{l}\mathrm{a}\mathrm{t}\mathrm{e}\mathrm{d}}-{P}_{\mathrm{C}\mathrm{O},i}^{\mathrm{U}\mathrm{N}\mathrm{e}\mathrm{t}1\mathrm{D}\mathrm{-}\mathrm{e}\mathrm{s}\mathrm{t}\mathrm{i}\mathrm{m}\mathrm{a}\mathrm{t}\mathrm{e}\mathrm{d}}\right)}^{2},$$

and

12 $${\mathrm{A}\mathrm{E}\mathrm{P}}_{i}=\mid {P}_{\mathrm{C}\mathrm{O},i}^{\mathrm{P}\mathrm{r}\mathrm{e}\mathrm{-}\mathrm{s}\mathrm{i}\mathrm{m}\mathrm{u}\mathrm{l}\mathrm{a}\mathrm{t}\mathrm{e}\mathrm{d}}-{P}_{\mathrm{C}\mathrm{O},i}^{\mathrm{U}\mathrm{N}\mathrm{e}\mathrm{t}1\mathrm{D}\mathrm{-}\mathrm{e}\mathrm{s}\mathrm{t}\mathrm{i}\mathrm{m}\mathrm{a}\mathrm{t}\mathrm{e}\mathrm{d}}\mid ,$$

where ${P}_{\mathrm{C}\mathrm{O},i}^{\mathrm{P}\mathrm{r}\mathrm{e}\mathrm{-}\mathrm{s}\mathrm{i}\mathrm{m}\mathrm{u}\mathrm{l}\mathrm{a}\mathrm{t}\mathrm{e}\mathrm{d}}$denotes the ground-truth clean CO pressure value prior to noise addition, and ${P}_{\mathrm{C}\mathrm{O},i}^{\mathrm{U}\mathrm{N}\mathrm{e}\mathrm{t}1\mathrm{D}\mathrm{-}\mathrm{e}\mathrm{s}\mathrm{t}\mathrm{i}\mathrm{m}\mathrm{a}\mathrm{t}\mathrm{e}\mathrm{d}}$represents the pressure predicted from the denoised spectra. No averaging over the *N*samples is applied, so ${\mathrm{S}\mathrm{E}\mathrm{P}}_{i}$ and ${\mathrm{A}\mathrm{E}\mathrm{P}}_{i}$ represent pressure estimation errors. In the mid-pressure range (0.3–0.5 to 1.75 bar), both errors become negligibly small. Outside this interval, the errors remain very low, with SEP on the order of 10^− 6^ – 10^− 4^ and AEP around 10^− 3^, indicating reliable pressure retrieval from the reconstructed Voigt profiles. To further evaluate the model’s generalization capability, additional test points at 2.5 bar and 3 bar, which lie outside the training range (0.01–2.2 bar), were also examined. The results show only a moderate increase in error under this extrapolation scenario, while the predicted pressures remain in close agreement with the true values, indicating stable model behavior beyond the training distribution. These results indicate that the model has not merely memorized a constrained mapping within the training interval but has learned a representation that generalizes to higher-pressure profiles.

**Fig. 7:**
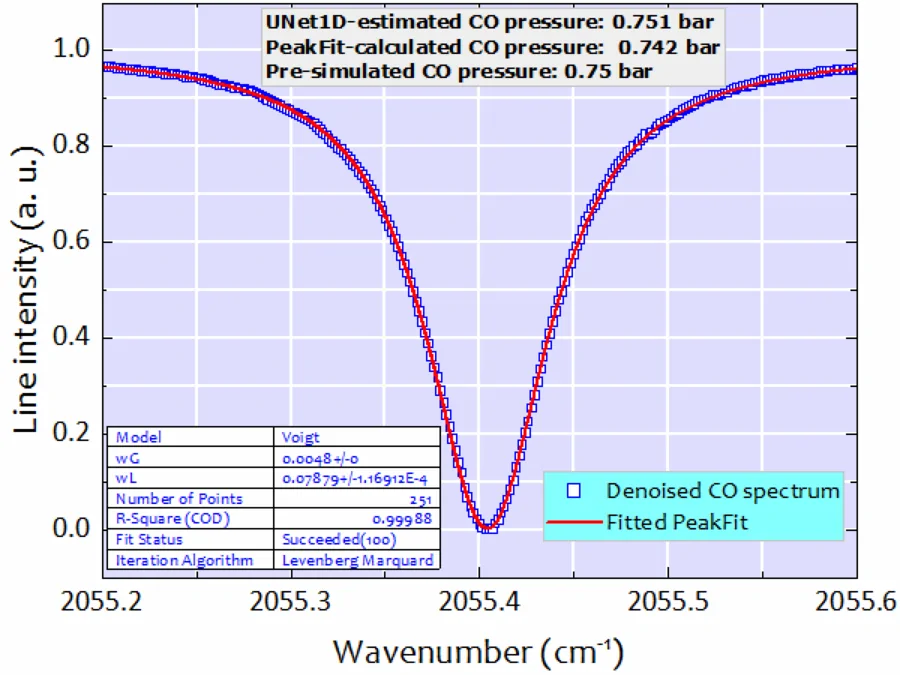
illustrates the Voigt profile fitting for a CO spectrum simulated at 750 mbar. The spectrum, extracted from a noise-contaminated signal, is denoised in the first stage of the 1D U-Net model (see Fig. 1). The inset table lists the Gaussian (w₍G₎) and Lorentzian (w₍L₎) widths as the profile parameters. The estimated pressures obtained from the 1D U-Net and PeakFit methods are shown at the top for direct comparison.

To further validate the ML-based CO pressure estimation, a selected subset of the 200 denoised spectra produced by the first stage of the 1D U-Net was analyzed using PeakFit, enabling a quantitative comparison with the pressures predicted by the network. A representative fitting result for 750 mbar is shown in Fig. 7.

The regression model predicted 0.751 bar, while curve fitting yielded 0.742 bar, both in close agreement with the pre-simulated pressure of 0.75 bar. The fit achieved R² = 0.99988, confirming the high accuracy and stability of the denoising stage and effective feature preservation in the joint U-Net–Pressure two-head model.

Table 3 presents a quantitative comparison for a subset of the 200 pre-simulated CO pressure test lines, estimated using the PeakFit method and the 1D U-Net model from the corresponding denoised CO spectra. The 1D U-Net pressure estimates are obtained directly from the denoised spectral data, without applying any additional analytical fitting procedures.

**Table 3:** Comparison of CO pressure estimates from denoised spectral lines using the PeakFit method and the 1D U-Net model.

Pre-simulated CO pressure (bar)	1D U-Net-estimated (bar)	PeakFit-calculated (bar)	Error of PeakFit	
			AEP	SEP
0.01	0.0183	0.0305	2.0 × 10^− 2^	4.20 × 10^− 4^
0.25	0.2496	0.2485	1.5 × 10^− 3^	2.25 × 10^− 6^
0.50	0.5003	0.4924	7.6 × 10^− 3^	5.77 × 10^− 5^
0.75	0.7524	0.7424	7.6 × 10^− 3^	5.77 × 10^− 5^
1.00	1.0005	0.9877	1.2 × 10^− 2^	1.51 × 10^− 4^
1.25	1.2500	1.2428	7.2 × 10^− 3^	5.18 × 10^− 5^
1.50	1.5091	1.5009	9.0 × 10^− 4^	8.10 × 10^− 7^
1.75	1.7524	1.7590	9.0 × 10^− 3^	8.10 × 10^− 5^
2.00	2.0131	2.0171	1.7 × 10^− 2^	2.92 × 10^− 4^

The SEP (Square Error of Pressur) and AEP (Absolute Error of Pressure) values were calculated to quantify the accuracy of the pressures obtained using the PeakFit method.

As can be seen from Table 3, the pressures estimated by the 1D U-Net model remain strongly correlated with those obtained using the PeakFit method over the entire 0–2 bar range. The deviations between the two approaches are consistently sub-percent, confirming the robustness of the proposed framework.

For example, at a pressure of 0.75 bar, the AEP is approximately 7.6 × 10^− 3^ bar, with a corresponding SEP of 5.77 × 10^− 5^ bar, demonstrating both linear agreement and physical interpretability of the denoised spectral outputs. Similarly, at intermediate and higher pressures (e.g., 1.50 bar), the errors further decrease, with AEP values below 10^− 4^ bar, highlighting the stability of the learned representation across varying pressure regimes.

These results confirm that the shared encoder features effectively preserve the quantitative spectral line-shape information required for accurate pressure retrieval, even in the presence of significant noise at low pressures. Moreover, the proposed model achieves an accuracy comparable to conventional spectroscopic fitting techniques, while substantially outperforming traditional PeakFit-based software in computational efficiency, making it well suited for real-time or large-scale spectroscopic applications.

### 1D U-Net model capability in denoising and pressure estimation: experiment

**Fig. 8:**
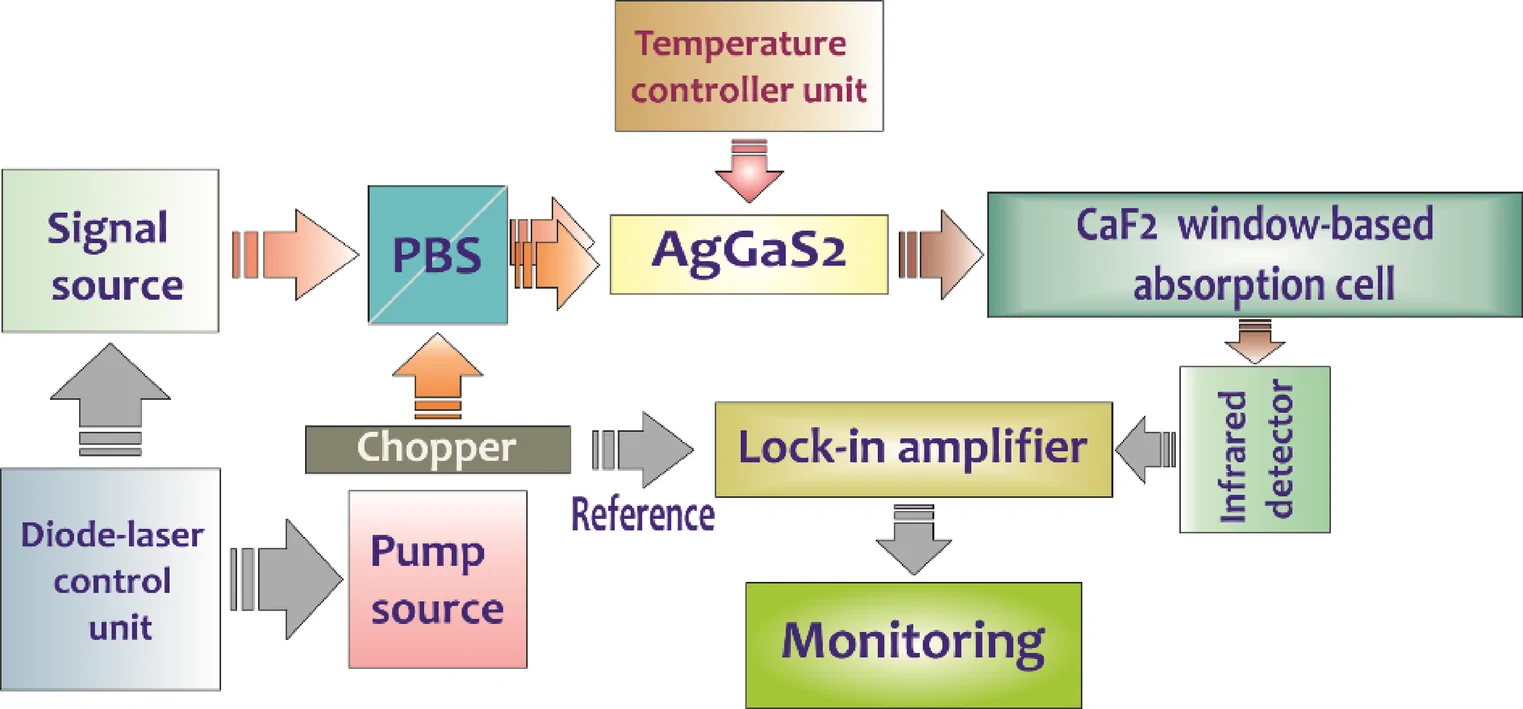
Schematic top-view of the DFG experimental setup used to generate coherent mid-infrared radiation tunable around the P(21) absorption line of CO. A mechanical chopper and a lock-in amplifier constitute the phase-sensitive detection system employed to isolate the weak DFG signal from background noise. The PBS is a polarizing beam splitter designed to collinearly align the pump and signal beams.

An experimental study utilizing a DFG-based spectroscopy system, depicted schematically in Fig. 8, investigates the effectiveness of the 1D U-Net model in estimating the gas pressure by analyzing the denoised spectrum of the P(21) CO absorption line.

The DFG setup depicted in Fig. 8^22^ employs a 680 nm, 30 mW pump diode laser (Toshiba TOLD9150) featuring a slope efficiency of 0.31 mW/mA and a tuning range of 0.075 nm, alongside a 790 nm, 20 mW signal laser (Sharp LT024MDO) with a slope efficiency of 0.11 mW/mA and a tuning range of 0.33 nm. To maximize nonlinear interaction, both pump and signal beams are circularized using anamorphic optics and combined collinearly via a polarizing beam splitter (PBS) before being focused into a 30-mm AgGaS₂ (AGS) nonlinear crystal, configured for type-I noncritical (e + o → o) phase matching. The crystal temperature was precisely stabilized to ± 0.1 °C and tuned between 27 °C and 81 °C, enabling MIR radiation generation spanning 4.8 to 5.1 μm with a spectral bandwidth of 3.6 cm⁻¹, corresponding to a temperature bandwidth of approximately 2.55 °C. The MIR radiation was isolated using a 2-mm cutoff filter and detected by an N₂-cooled InSb detector (Polytec R-1703-IS) with a calibrated sensitivity of 5.277 nW/V. To suppress optical and electrical noise and thereby improve the SNR, a mechanical chopper in combination with lock-in detection is employed. Under these operating conditions, a DFG output power of approximately 29 nW is obtained, corresponding to a conversion efficiency of 0.06 mW W⁻² cm⁻¹ and a linewidth narrower than 1.2 × 10⁻² cm⁻¹.

**Fig. 9:**
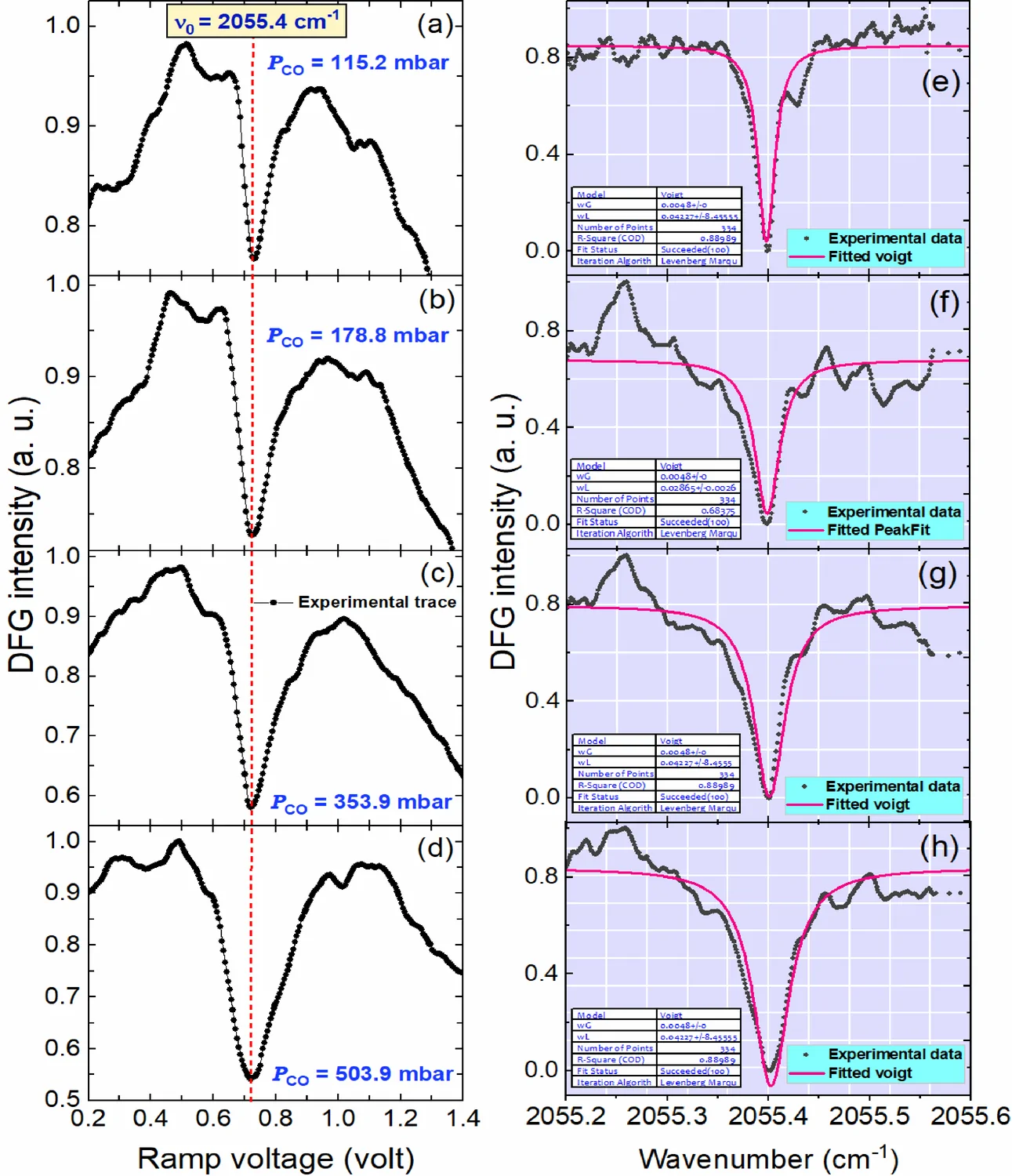
Experimental traces of the P(21) CO absorption line obtained using the DFG setup shown in Fig. 7. The absorption cell was filled with pure CO, with the pressure controlled to within ± 0.1 mbar. Traces (**a**)–(**d**) show the raw signals at four different CO pressures as the DFG wavelength was scanned across the line center using a low-frequency sawtooth ramp. The AGS crystal was phase-matched at 28.1 ± 0.1 °C, while the lock-in amplifier operated with a sensitivity of 30 mV and a time constant of 300 ms, yielding a detection sensitivity of 1.5 10⁻⁴ at a chopper frequency of ~ 540 Hz. Traces (**e**)–(**h**) present the corresponding background-corrected spectra plotted versus wavenumber, calibrated using a Fabry–Perot etalon to enable accurate PeakFit analysis. The slopes observed in traces (**a**)–(**c**) originate from DFG intensity variations with the ramp voltage.

CO detection was performed using a 10 cm single-pass cell filled with pure CO gas. The DFG wavelength was tuned by applying a low-frequency current ramp to the signal diode laser while keeping the pump laser fixed. The AGS crystal temperature was stabilized at 27.9 ± 0.1 °C to maintain phase matching and maximize the DFG output intensity. The MIR-DFG radiation was scanned over a narrow spectral window of approximately 1.1 cm⁻¹ around the P(21) CO absorption line using a 5 V sawtooth ramp, ensuring spectral isolation from neighboring CO transitions and other interfering species. The CO gas was maintained at room temperature to minimize thermal effects and emphasize pressure-dependent spectral variations. This measurement procedure was repeated for sixteen distinct CO pressures. Figure 9 presents representative experimental spectra of the CO absorption line measured at four selected pressures, with the cell pressure controlled within ± 0.1 mbar.

Although minor variations appear in the outer wings of Fig. 9a–d, the central region remains stable, clean, and noise-free. This consistency indicates that the signal fidelity is maintained despite fluctuations in the ramped drive voltage during the pressure-dependent DFG scan. Data obtained from Fig. 8 regarding the CO pressure initially introduced into the cell and the corresponding values estimated via the PeakFit software are presented in Table 4.

**Table 4:** CO pressures derived from background-corrected experimental traces in Fig. 8e–h using the PeakFit software, together with the AE and SE values assessing the consistency between the estimated and experimental pressures.

Pre-filled experimental CO pressure (bar)	PeakFit-estimated CO pressure (bar)	Error	
		AE	SE
0.1152	0.180	0.065	0.0042
0.1788	0.269	0.090	0.0164
0.3539	0.341	0.012	0.0145
0.5039	0.398	0.105	0.0110

As presented in Table 4, although the differences between the two CO pressures quantified by the AE and SE values are small, the pressure estimated by PeakFit remains slightly offset from the pre-filled experimental value. Among the four examined pressures, the CO pressure of 353.9 ± 0.1 mbar shows the closest agreement with the PeakFit-estimated value, exhibiting the lowest AE of 0.012.

### Denoising procedure

**Fig. 10:**
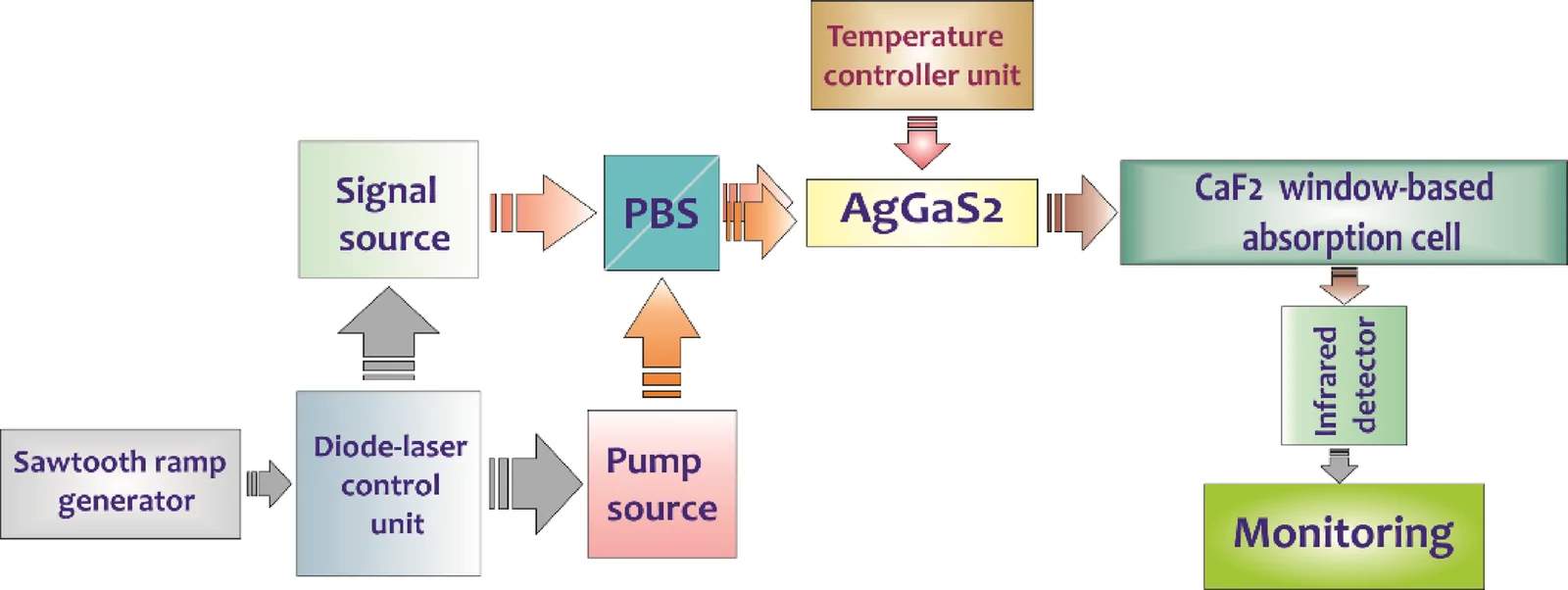
Phase-sensitive detection components, including the mechanical chopper and lock-in amplifier, were removed from the DFG setup shown in Fig. 8. This configuration was used to acquire a spectrum containing the P(21) CO absorption line, which becomes indistinguishable within the background noise.

As shown in Fig. 10, to evaluate the denoising capability of the 1D U-Net model on the experimental P(21) CO spectral line, the chopper and lock-in amplifier were removed from the DFG setup. Under identical experimental conditions, eliminating these components reintroduced background noise that had previously been suppressed. Consequently, the CO spectral feature became indistinguishable from the unfiltered noise background, resulting in a measured spectrum composed of both signal and noise, with the CO line almost entirely obscured.

**Fig. 11:**
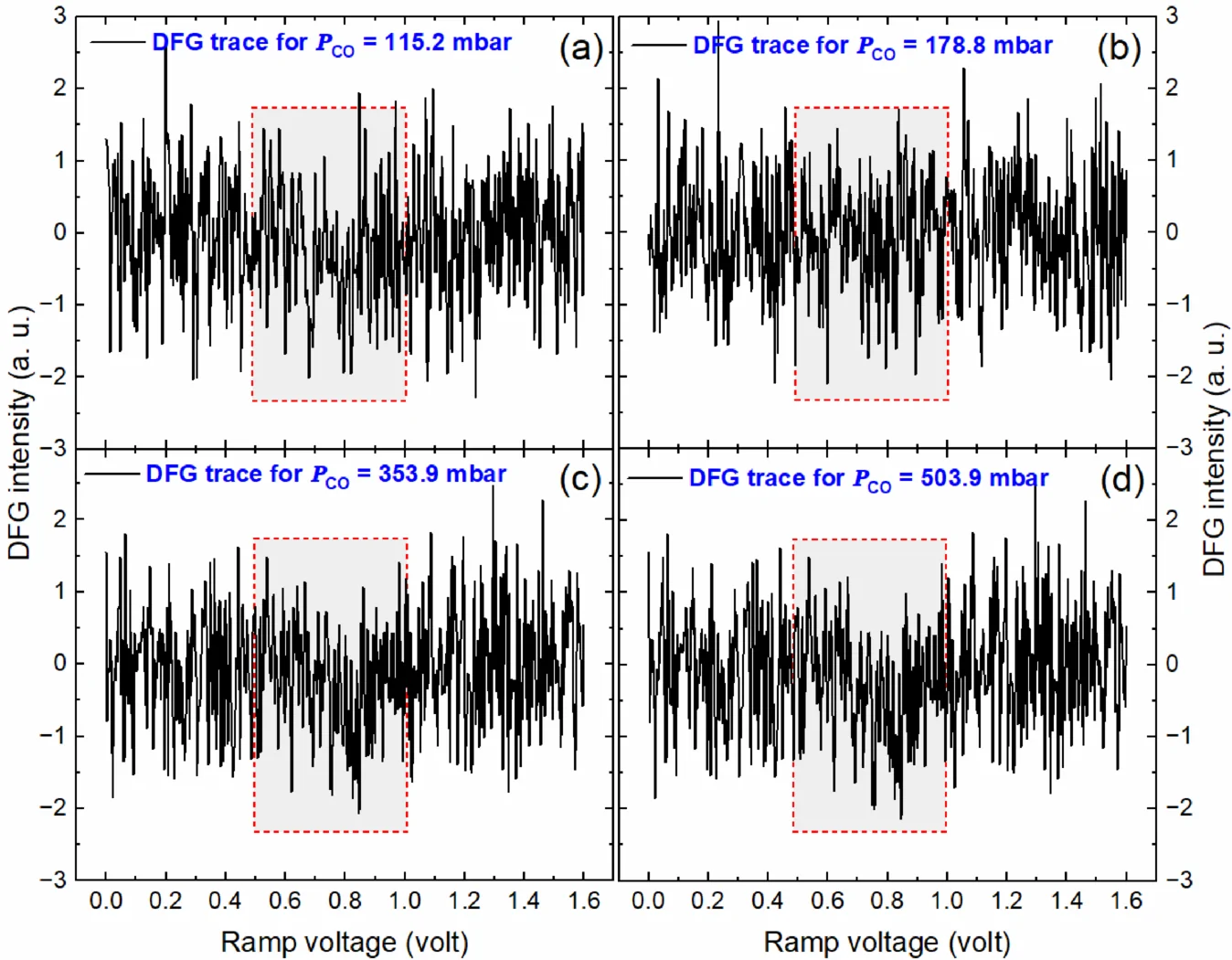
DFG traces containing the CO spectra recorded for four representative gas pressures presented in Fig. 9. The measurements were conducted using the experimental setup shown in Fig. 10, operated without the lock-in amplifier and mechanical chopper. Identical measurement procedures under comparable technical conditions were applied to all 16 CO pressure levels. The dashed red rectangles highlight the region of the ramp voltage where CO absorption is expected to take place.

The generated DFG beam was scanned across the target transition while propagating through the CO absorption cell. In this configuration, the mechanical chopper and lock-in amplifier were intentionally omitted to allow direct detection of the CO absorption signal. As a result, the recorded spectra contained unfiltered noise due to the absence of modulation and lock-in detection. This measurement procedure was repeated for sixteen different CO pressure levels, and all spectra were recorded for subsequent analysis. Figure 11 shows representative traces corresponding to the four CO pressures presented in Fig. 9.

**Fig. 12:**
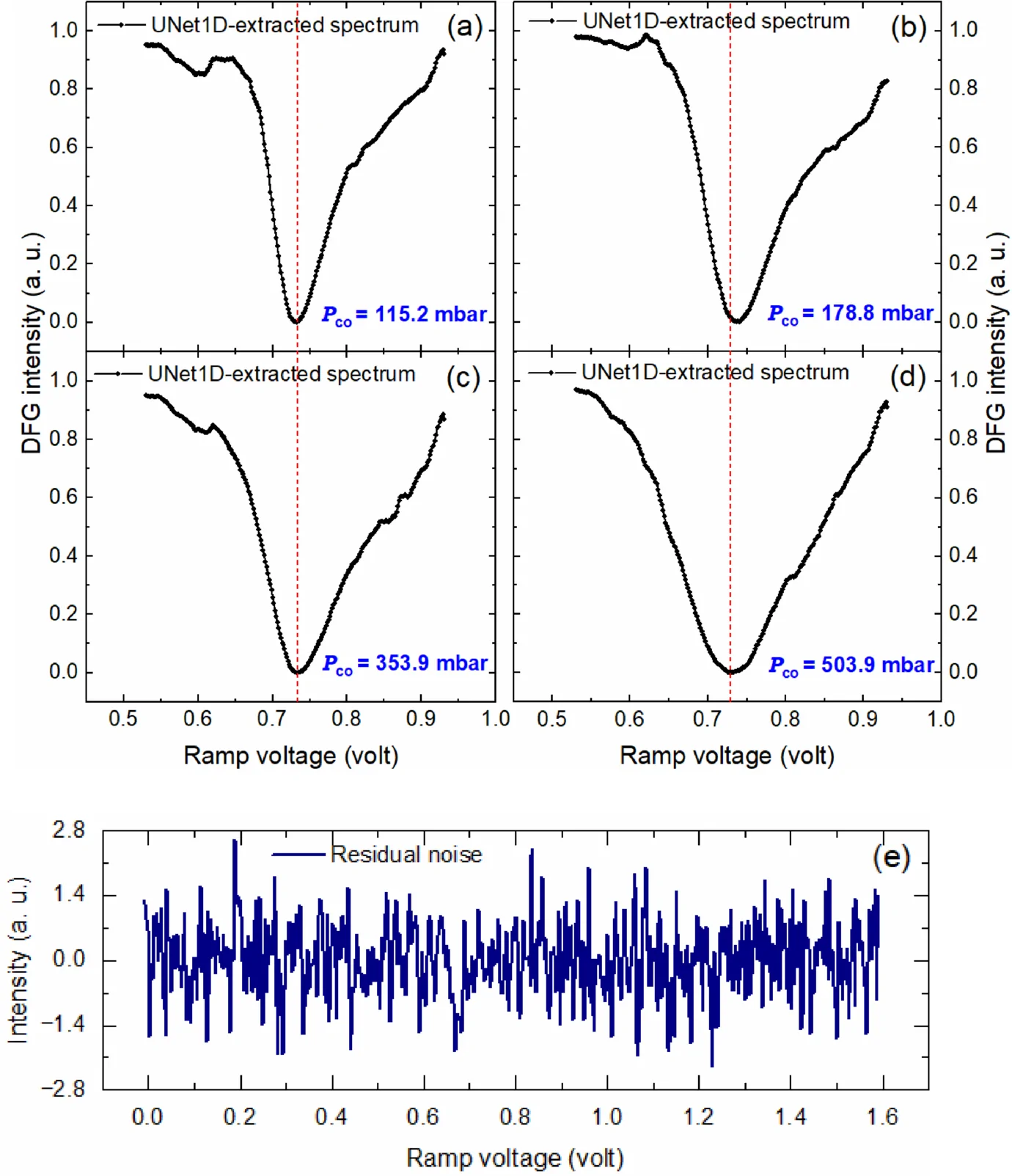
CO absorption Voigt spectra extracted from the DFG traces shown in Fig. 11, obtained using the experimental setup depicted in Fig. 10 without employing the phase-sensitive detection system. Representative spectra are presented for four CO gas pressures: (**a**) 115.2 mbar, (**b**) 178.8 mbar, (**c**) 353.9 mbar, and (**d**) 503.9 mbar, selected from the 16 different pressure values. An example of the residual noise spectrum obtained after denoising is shown in panel (**e**).

As shown in Fig. 11, the recorded DFG spectra containing the CO absorption lines at four different pressures exhibit pronounced fluctuations relative to the signal depths presented in Fig. 9. These variations stem from the absence of a phase-sensitive detection device, which limits the ability to discriminate the weak CO absorption features from background noise. However, when the CO pressure is raised from 115.2 to 503.9 mbar, the absorption spectrum emerges more distinctly within the noise. Figure 12 highlights the capability of the 1D U-Net model to accurately extract CO spectra from the experimentally measured DFG scans depicted in Fig. 11.

**Fig. 13:**
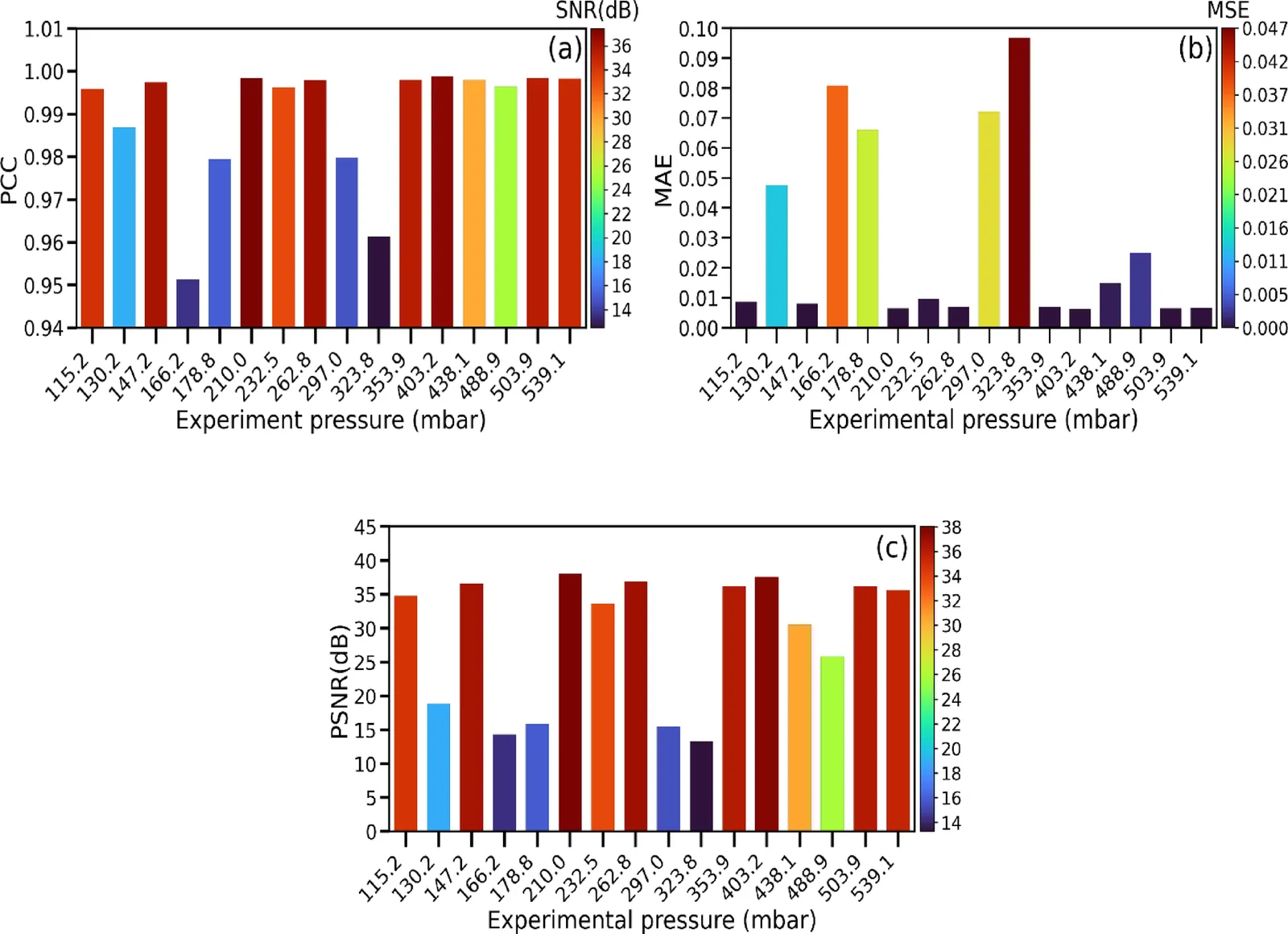
Quantitative evaluation of the 1D U-Net model ability in extracting the CO spectra from the DFG scans performed based on the setup schematically characterized in Fig. 10 for 16 CO experimental pressures ranging from 115.2 mbar to 539.1 mbar. (**a**) Indicates the structural fidelity assessment using PCC represented by bar height, with SNR mapped to the color scale, (**b**) is the error analysis displaying MAE and MSE, and (**c**) characterizes the PSNR distribution indicating reconstruction quality.

As shown in Fig. 12, the extracted CO spectra closely match those presented in Fig. 9a–d. A negligible line-center shift of approximately 0.007 V is observed, which is negligible compared to the linewidth. Figure 13 summarizes the performance of 1D U-Net in extracting all 16 experimental CO pressures from the noise spectra, evaluated using the standard introduced metrics.

The experimental results demonstrate that the proposed model achieves high-fidelity reconstruction across most of the pressure range. As shown in Fig. 13a, c, the denoised signals exhibit strong structural agreement with the ground truth, with PCC values consistently above 0.99 and PSNR exceeding 34 dB for nearly 70% of the test cases. The model performs best at stable pressure points such as 210.0 mbar, where it reaches a maximum PSNR of 38.01 dB and a minimum MSE of 1.58 × 10⁻⁴, indicating effective noise suppression while preserving key spectral features.

Despite the overall stability, the error analysis in Fig. 13b reveals distinct nonlinear performance fluctuations at specific pressure intervals. A notable degradation in signal quality is observed at 166.2 mbar and 323.8 mbar, where the MAE peaks at roughly 0.097 and the SNR drops to its lowest value of 12.48 dB. However, even in these lower-performance regimes, the model maintains a correlation coefficient above 0.95, suggesting that the degradation is primarily due to amplitude scaling errors rather than a loss of structural pattern. Overall, the model proves to be highly effective, delivering reliable denoising performance for the vast majority of the experimental domain.

### Pressure estimation

Given the strong performance of the 1D U-Net model in extracting CO absorption spectra from noisy DFG scans, the model was further applied to estimate the line pressures directly from the denoised spectra. Four representative examples are shown in Fig. 11a–d. In of approach, the model infers the pressure of each spectral line using only the denoised spectra of the 16 CO measurements, relying solely on the Voigt line shapes without requiring additional analytical evaluation of the linewidth, as typically needed in PeakFit analysis.

A key distinction of this approach from our previous study^22^ is that pressure estimation is achieved without using background-corrected spectra and without calibrating the ramp voltage to wavenumber, a procedure that is often challenging in practical spectroscopic analysis. For comparison, the pressures of all 16 experimental CO lines were also determined from their background-corrected spectra using PeakFit.

**Fig. 14:**
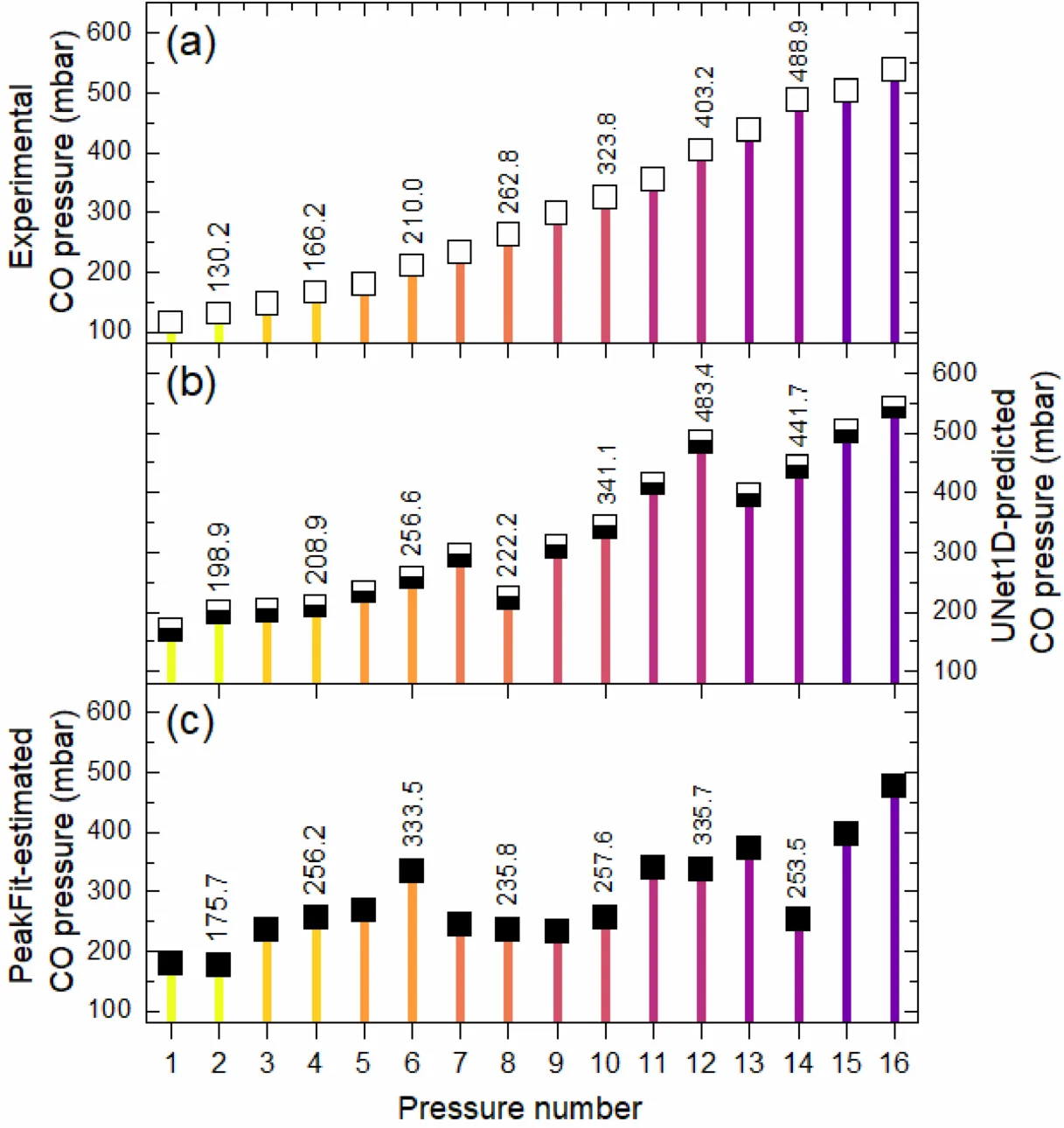
Comparison of CO pressures obtained from the experimental measurements (**a**), 1D U-Net predictions (**b**), and PeakFit estimates (**c**) for all 16 pressure samples. PeakFit values are derived from background-corrected spectra of the CO lines shown in Fig. 9a–d, whereas the 1D U-Net predictions are based solely on the denoised CO spectral profiles illustrated as examples in Fig. 11a–b.

The comparison in Fig. 14 demonstrates the ability of the 1D U-Net model to reconstruct the pressure trend while suppressing experimental noise. As shown in Fig. 14a, the experimental pressures increase nearly linearly, and Fig. 14b shows that the pressures predicted by the 1D U-Net reproduce the same overall trend with a smooth progression. In contrast, Fig. 14c exhibits larger deviations, mainly due to manual peak-fitting uncertainties and subjective calibration. The irregular variations between neighboring points indicate human-induced errors and the limitations of the time-consuming fitting procedure. Overall, both visual and numerical comparisons indicate that the 1D U-Net model provides higher accuracy, better reproducibility, and faster processing than the conventional manual fitting approach, making it a more efficient method for gas-pressure estimation.

**Fig. 15:**
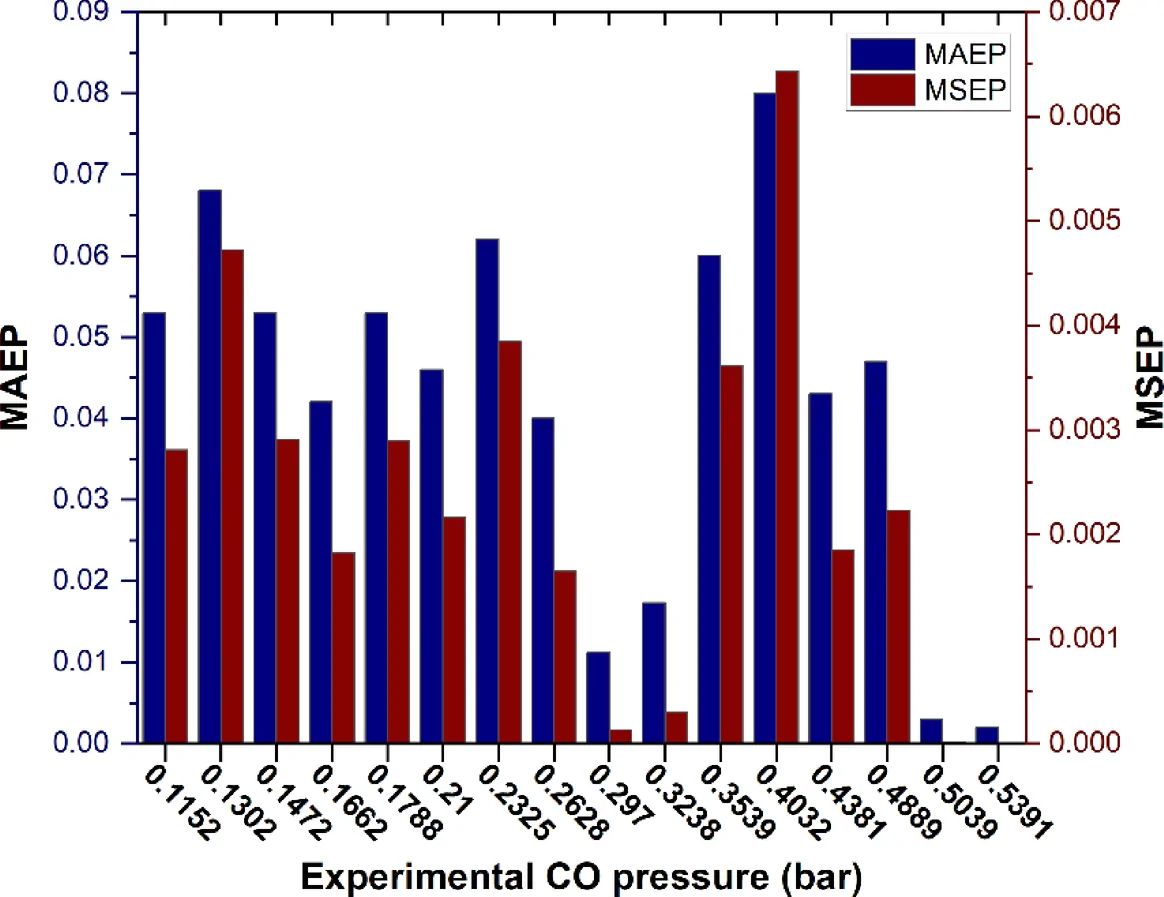
Correlation between experimental and predicted CO pressures obtained through calculating the MAEP and MSEP values for each CO pressure.

Figure 15 further summarizes the predictive behavior of the 1D U-Net model using MAEP and MSEP metrics, which quantify how closely the predicted pressures match the corresponding reference values across the entire pressure range.

The corresponding pressure prediction errors inferred from Fig. 15, quantified by the MAEP and MSEP metrics, are summarized in Table 5.

**Table 5:** Comparison between experimentally measured and 1D U-Net-predicted CO pressures, alongside the AEP and SEP. The dataset validates the predictive performance of the proposed model for quantitative CO pressure estimation.

Experimental pressure (bar)	Predicted pressure (bar)	Error		Experimental pressure (bar)	Predicted pressure (bar)	Error	
		AEP	SEP			AEP	SEP
0.1152	0.1682	0.053	2.81 × 10^− 4^	0.2970	0.3082	0.011	12.5 × 10^− 5^
0.1302	0.1989	0.068	47.2 × 10^− 4^	0.3238	0.3411	0.017	29.9 × 10^− 5^
0.1472	0.2011	0.053	29.1 × 10^− 4^	0.3539	0.4140	0.060	36.1 × 10^− 4^
0.1662	0.2089	0.042	18.2 × 10^− 4^	0.4032	0.4834	0.080	6.40 × 10^− 3^
0.1788	0.2326	0.053	28.9 × 10^− 4^	0.4381	0.3951	0.043	18.4 × 10^− 4^
0.2100	0.2565	0.046	21.6 × 10^− 4^	0.4889	0.4417	0.047	22.0 × 10^− 4^
0.2325	0.2945	0.062	38.4 × 10^− 4^	0.5039	0.5008	0.003	9.00 × 10^− 6^
0.2628	0.2222	0.040	16.5 × 10^− 4^	0.5391	0.5411	0.002	4.00 × 10^− 6^

The results reported in Table 5 confirm that the 1D U-Net-predicted values closely follow the experimental trend, with minor deviations across the range. The average MAEP and MSEP are 0.0227 and 1.38 × 10⁻³, respectively, indicating strong consistency between the model predictions and physical measurements. However, the lowest error is observed at a CO pressure of 539.1 mbar with an AEP and an SEP of 0.002 and 4 × 10⁻⁶, respectively, whereas the highest errors occur at 403.2 mbar with an AEP of 0.08 and an SEP of 6.4 × 10⁻³. The 1D U-Net model, therefore, maintains sub-0.03 bar typical accuracy across the operating range, validating its robustness for precise CO quantification. At some pressures, the spectral features become comparable to the experimental noise level or the absorption line exhibits lower pressure sensitivity, which naturally increases the retrieval uncertainty for those operating points. These localized deviations reflect the underlying measurement physics rather than any inconsistency in the proposed model.

#### Sensitivity Analysis of Loss Weighting (λ)

To examine whether the shared-encoder architecture suffers from multitask interference between reconstruction and pressure estimation, we evaluated the effect of varying the regression loss weighting factor. Four settings were tested to quantify how the balance of gradient contributions influences denoising quality and pressure estimation accuracy. The results of this sensitivity analysis are summarized in Tables 6 and 7.

**Table 6:** Pressure estimation of model under different conditions.

Pressure weight, λ	Actual pressure (bar)	Estimated pressure (bar)	AE
0.1	0.20	0.199	0.001
	1.20	1.199	0.001
	1.80	1.810	0.010
0.2	0.20	0.198	0.002
	1.20	1.199	0.001
	1.80	1.807	0.007
0.5	0.20	0.197	0.003
	1.20	1.202	0.002
	1.80	1.812	0.012
1.0	0.20	0.191	0.009
	1.20	1.197	0.003
	1.80	1.804	0.004

**Table 7:** Denoising performance of model under different conditions.

Pressure (bar)	Pressure weight, λ	SNR (dB)	MSE
0.2	0.1	35.03	3.04 × 10^− 4^
	0.2	44.85	3.16 × 10^− 5^
	0.5	37.98	1.54 × 10^− 4^
	1.0	33.12	4.72 × 10^− 4^
1.2	0.1	42.58	4.62 × 10^− 5^
	0.2	52.64	4.55 × 10^− 6^
	0.5	43.24	3.97 × 10^− 5^
	1.0	39.29	9.86 × 10^− 5^
1.8	0.1	42.56	4.27 × 10^− 5^
	0.2	48.85	1.00 × 10^− 5^
	0.5	42.82	4.02 × 10^− 5^
	1.0	39.66	8.33 × 10^− 5^

Overall, the results indicate that *λ* = 0.2 offers the most effective balance between reconstruction fidelity and regression accuracy. Higher values introduce noticeable degradation in denoising performance, while lower values do not improve pressure estimation robustness. Therefore, *λ* = 0.2 was selected as the optimal configuration for joint training of the two tasks.

### Robustness to instrumental drift

In practical spectroscopic measurements, slow instrumental fluctuations such as changes in laser power slope or deviations from ideal ramp linearity may occur over time. These effects can introduce baseline tilts or distortions in the voltage–frequency mapping of the scan and may influence the stability of pressure retrieval algorithms. To evaluate the robustness of the proposed machine-learning framework under such conditions, we performed a controlled perturbation analysis using the pseudo-experimental spectra employed during the fine-tuning stage. Two types of instrumental drift were simulated: (i) baseline tilt representing variations in the laser power slope across the scan, and (ii) ramp nonlinearity representing distortions in the DFG sawtooth ramp. For each case, perturbation levels of 5% and 10% relative to the nominal configuration were applied. The trained model was then used to predict pressure values from these perturbed spectra without any additional retraining. The resulting predictions for several representative experimental pressures are summarized in Table 8.

**Table 8:** Predicted pressure under controlled drift conditions.

True experimental pressure (bar)	Predicted pressure (bar)				
	No Tilt	Baseline Tilt		Ramp Drift	
	0%	5%	10%	5%	10%
0.1189	0.1521	0.1691	0.1795	0.1721	0.1721
0.3238	0.3267	0.3413	0.3577	0.3689	0.3686
0.5391	0.5910	0.5812	0.5690	0.5901	0.5881

The results indicate that increasing drift levels produce only moderate shifts in the predicted pressure values. Even at the highest perturbation level (10%), the model predictions remain well-behaved and do not exhibit unstable or irregular behavior. Although a small systematic bias appears as the drift increases, the overall response remains smooth and predictable under both baseline tilt and ramp distortion conditions. These findings suggest that the learned spectral representation retains robustness against common instrumental fluctuations encountered in real experimental environments. Consequently, the proposed ML-based framework demonstrates practical stability for pressure estimation even when moderate ramp or power-slope variations occur during measurements.

### Temperature sensitivity analysis

In practical sensing environments, small temperature fluctuations may influence spectral line shapes through Doppler broadening. Since the present model was trained using spectra generated at a constant temperature of 296 K, it is important to evaluate the sensitivity of the pressure retrieval to realistic temperature variations.

To investigate this effect, a perturbation analysis was conducted by generating Voigt profiles at temperatures of 293 K, 296 K, and 299 K for several representative pressures (0.10, 0.75, 1.25, and 2.00 bar). These spectra were then processed by the trained model without retraining. Table 9 summarizes the predicted pressures and absolute errors.

**Table 9:** Temperature sensitivity analysis of pressure retrieval.

True experimental pressure (bar)	Temperature (k)	Predicted pressure (bar)	AE (bar)
0.10	293	0.121	0.021
	296	0.092	0.008
	299	0.092	0.008
0.75	293	0.752	0.002
	296	0.748	0.002
	299	0.737	0.013
1.25	293	1.248	0.002
	296	1.250	0.000
	299	1.251	0.001
2.0	293	2.000	0.000
	296	2.004	0.004
	299	1.995	0.005

The results show that the model maintains stable performance across the tested temperature range. Although the Doppler FWHM changes slightly with temperature (approximately 0.00475–0.00485 cm⁻¹ across 293–299 K), the resulting pressure estimates remain highly accurate. The maximum deviation observed in this analysis is only a few hundredths of a bar, indicating that the proposed framework is robust against typical laboratory or field temperature drifts (± 3 K).

## Conclusion

This study presented a new 1D U-Net architecture that integrates spectral denoising and pressure estimation of the P(21) CO absorption line at 2055.4 cm⁻¹ within a unified, end-to-end machine-learning framework. The model’s performance was rigorously validated using both simulated and experimental data.

During the simulation phase, the model was trained on 2000 synthesized Voigt profiles corresponding to pressures between 1 mbar and 2 bar with 0.01 mbar resolution. After training, the model denoised 200 unseen, noise-contaminated experimental spectra and directly estimated their pressures using only the spectral inputs—without any auxiliary fitting software or conventional analytical routines. Quantitative evaluation confirmed the model’s robustness: denoised spectra exhibited Pearson correlation coefficients (PCC) close to 1. As summarized in Table 2, the model achieved its minimum error at 1.25 bar, where the prediction error was essentially zero (AEP ≈ 0, SEP ≈ 0). The largest deviation occurred at 3.00 bar with AEP = 5.41 × 10⁻² and SEP = 2.93 × 10⁻³.

The model was further validated using 16 experimental CO absorption lines recorded at different pressures. These spectra were initially acquired with a phase-sensitive DFG-based spectrometer scanning around the line center. To challenge the model, the DFG source was subsequently re-scanned without the lock-in amplifier and mechanical chopper, producing traces in which the CO absorption signal was nearly indistinguishable within a high-noise background.

To mitigate this limitation, a specialized instance of the model was fine-tuned using a pseudo-experimental dataset. Robustness analyses under controlled ramp and baseline perturbations further demonstrate that the proposed framework maintains stable performance under realistic instrumental variations.

The 1D U-Net model successfully recovered clean CO spectra from noisy measurements, achieving a PCC above 0.99 and a PSNR exceeding 34 dB for more than 70% of the DFG traces. The denoised spectra were then directly used for pressure retrieval using the proposed adaptive inference strategy, which automatically switches to fine-tuned weights and operates in the raw ramp-voltage domain without requiring manual background correction or wavenumber calibration. The sensitivity analysis also confirmed that *λ* = 0.2 provides a stable balance between reconstruction fidelity and pressure estimation, without introducing notable multitask interference.

The pressures predicted by the 1D U-Net model showed strong agreement with the known fill pressures of the CO cell. Across all 16 spectra, the prediction errors remained low, with the maximum deviation observed at 403.2 mbar, corresponding to an AEP of 0.08 and an SEP of 6.4 × 10⁻³.

Collectively, the high PCC and PSNR values, together with the minimal pressure-estimation errors, confirm that the proposed model achieves both high-fidelity denoising and accurate physical parameter retrieval. The algorithm effectively suppresses noise while preserving the intrinsic pressure-dependent line shapes of the absorption spectra. Its consistent performance over a wide pressure range highlights strong generalization capability and suitability for practical laser-based gas sensing in variable-pressure environments. The small deviations observed in pressure prediction can be attributed to several factors. First, minor distortions introduced during the denoising stage may propagate to the regression step, producing slight discrepancies. Second, because the initial model was primarily trained on simulated data due to the limited availability of experimental samples, subtle mismatches between the simulated ramp structure and complex real-world artifacts may remain, even after amplitude-based fine-tuning. In addition, device-specific distortions and varying experimental conditions mean that a single static model cannot achieve optimal accuracy across fundamentally different optical systems.

## Data Availability

Data underlying the results presented in this paper are not publicly available at this time but may be obtained from the authors upon reasonable request.

## Code availability

The custom code used for data simulation, model training, and inference in this study is available at:

https://github.com/alirezaraheemi/Multi-Task-Residual-U-Net-for-Simultaneous-Denoising-and-Gas-Pressure-Retrieval-from-Noisy-Spectra.git.

The repository includes the essential scripts required to reproduce the main results of the manuscript.
